# Mechanisms of microtubule dynamics and force generation examined with computational modeling and electron cryotomography

**DOI:** 10.1038/s41467-020-17553-2

**Published:** 2020-07-28

**Authors:** Nikita B. Gudimchuk, Evgeni V. Ulyanov, Eileen O’Toole, Cynthia L. Page, Dmitrii S. Vinogradov, Garry Morgan, Gabriella Li, Jeffrey K. Moore, Ewa Szczesna, Antonina Roll-Mecak, Fazoil I. Ataullakhanov, J. Richard McIntosh

**Affiliations:** 1 Department of Physics, Lomonosov Moscow State University, Moscow, Russia; 2 Center for Theoretical Problems of Physicochemical Pharmacology, Russian Academy of Sciences, Moscow, Russia; 3 Dmitry Rogachev National Research Center of Pediatric Hematology, Oncology and Immunology, Moscow, Russia; 4 Department of Molecular, Cellular, and Developmental Biology, University of Colorado, Boulder, CO USA; 5 Department of Cell and Developmental Biology, University of Colorado School of Medicine, Aurora, CO USA; 6 Cell Biology and Biophysics Unit, National Institute of Neurological Disorders and Stroke, Bethesda, MD USA

**Keywords:** Biochemistry, Computational biophysics, Molecular conformation, Supramolecular assembly, Kinetochores

## Abstract

Microtubules are dynamic tubulin polymers responsible for many cellular processes, including the capture and segregation of chromosomes during mitosis. In contrast to textbook models of tubulin self-assembly, we have recently demonstrated that microtubules elongate by addition of bent guanosine triphosphate tubulin to the tips of curving protofilaments. Here we explore this mechanism of microtubule growth using Brownian dynamics modeling and electron cryotomography. The previously described flaring shapes of growing microtubule tips are remarkably consistent under various assembly conditions, including different tubulin concentrations, the presence or absence of a polymerization catalyst or tubulin-binding drugs. Simulations indicate that development of substantial forces during microtubule growth and shortening requires a high activation energy barrier in lateral tubulin-tubulin interactions. Modeling offers a mechanism to explain kinetochore coupling to growing microtubule tips under assisting force, and it predicts a load-dependent acceleration of microtubule assembly, providing a role for the flared morphology of growing microtubule ends.

## Abstract (web summary)

Microtubules are dynamic tubulin polymers which elongate by addition of bent guanosine triphosphate tubulin to the tips of curving protofilaments. Here authors use Brownian dynamics modeling and electron cryotomography to show that the lateral activation energy barrier in tubulin-tubulin interactions is a key parameter for this process, controlling the development of high pulling forces.

## Introduction

Microtubules (MTs) are dynamic protein polymers found in the cytoplasm of almost all eukaryotic cells. They elongate by the polymerization of tubulin dimers bound to guanosine triphosphate (GTP). The bound GTP is then hydrolyzed to guanosine diphosphate (GDP), leading to conformational changes in tubulin that render the MT lattice less stable. When the MT tip contains a critical number or density of GDP-tubulins, MTs initiate rapid shortening, losing tubulin from their tips^1^. In cells, MTs’ “minus-ends” are usually stabilized by capping proteins^2^, but the “plus-ends” grow outward from initiation centers toward the cell periphery. Each MT shows stochastic transitions to the shortening state, allowing these polymers to explore the intracellular volume and filling it with an array of transport tracks that can affect cell shape and reposition organelles.

MT dynamics are particularly important during mitosis; they allow spindle MTs to search for and bind chromosomes^3^. Upon encountering a chromosome, MTs serve as force-generating machines, playing a major role in the segregation of DNA to daughter cells^4^. The key part of a chromosome for interactions with MTs is its kinetochore, a structure that governs chromosome interactions with the mitotic spindle and alters the dynamics of MTs that become bound to it. In species with few MTs per kinetochore, a multi-subunit protein complex called Dam1 or DASH encircles the MTs and holds on as they depolymerize^5,6^. This complex can also couple micro-cargos to MT tips shortening in vitro under significant opposing forces^7–11^. Assisting forces, acting through purified yeast kinetochores that contain Dam1 rings and MT polymerases, can accelerate MT elongation^12–14^, although this mechanism is not yet understood.

Our understanding of MT dynamics, force generation, and the coupling of cargo with growing and shortening MT tips is limited by our lack of detailed knowledge about MT tip structures. Evidence from early electron cryomicroscopy revealed mostly blunt ends on growing MTs and flared ends on shortening MTs^15^. This suggested an elegant model in which GTP-tubulin is straight so it can assemble, whereas GDP-tubulin is intrinsically bent, so its straight configuration in MTs requires stress. However, further EM studies have reported a range of shapes for growing MT ends: gently curved protofilament (PF) bundles (“tubulin sheets”) seen in vitro^16,17^, and flared or blunt tips, but not sheets on MTs growing in vivo^18–20^. Moreover, X-ray, SAXS and allo-colchicine binding experiments have challenged the classic model by showing that both GTP- and GDP-tubulins are bent in solution^21–24^. Our recent descriptions of MT tips by electron cryotomography (cryoET) in vivo and in vitro found flaring PFs at the ends of both growing and shrinking MTs^25^. Similar curved shapes of PFs at the ends of growing MTs have been also observed in two other cryoET studies^26,27^.

Curved PFs at MT tips may have profound implications for the mechanisms of MT dynamics, of mechanical force generation, and of the regulation of both processes by associated proteins. Here, we use Brownian dynamics modeling to unravel how curved PFs can be straightened by thermal fluctuations, yet tubulin addition and loss can still generate large pulling and pushing forces during MT shortening and growth. We show that the lateral activation energy barrier in tubulin−tubulin interactions is a key parameter, controlling the development of high pulling forces. It is also important in accurate accounting for the unevenness of PF behavior at MT ends. To test our model predictions, we use cryoET to examine MT tip shapes under a range of conditions, including the presence of a polymerization catalyst and tubulin-binding drugs. These analyses identify potential mechanisms for the regulation of MT assembly by associated proteins and drugs. Finally, our simulations provide a mechanism for load-dependent acceleration of MT assembly; they thereby suggest a role for flared MT tips in synchronizing MT growth and shortening rates during chromosome oscillations.

## Results

### Curved PFs straighten frequently and develop bending forces

**Fig. 1:**
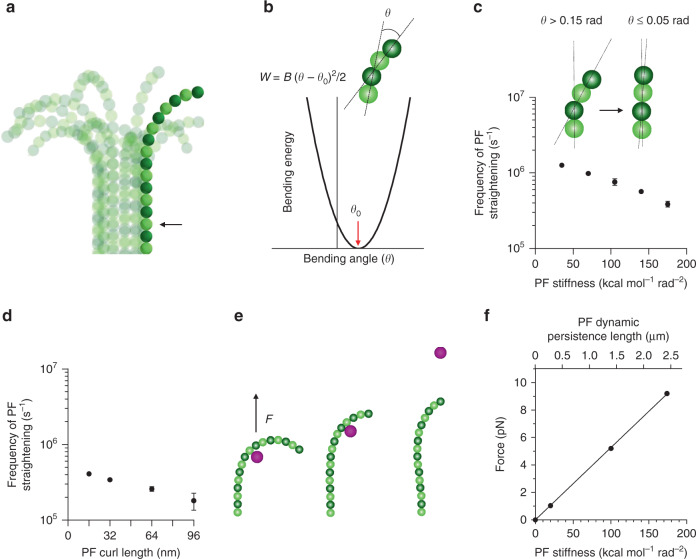
Brownian dynamics analysis of the properties of isolated tubulin PFs. **a** Visualization of a single PF. Darker and lighter spheres depict β- and α-tubulins, respectively. For illustration, tubulins in other PFs of the MT, which were not part of this simulation, are shown as semi-transparent spheres. The bottom tubulin dimer of the simulated PF is fixed in place, while the other tubulins experience Brownian fluctuations. **b** Potential energy of PF bending, described with the formula above the graph. **c** Predicted dependence of the frequency of PF straightening by thermal fluctuations on the PF bending stiffness. Straightening frequency is calculated as the number of passages per second from curved (*θ* > 0.15 rad) to straight (*θ* ≤ 0.05 rad) configurations at the lowest nonrestrained tubulin monomer in the PF (black arrow in **a**). Data represent mean ± s.d. from three repeats of each simulation. Source data are provided as a Source Data file. **d** Predicted dependence of the frequency of PF straightening by thermal fluctuations on the length of the PF. The frequency is scored as in panel **c**. Simulations were carried out with PF bending stiffness of 174 kcal mol^−1^ rad^−2^. Data represent mean ± s.d. from three repeats of each simulation. Source data are provided as a Source Data file. **e** A single PF experiencing an extensive force. In this case, the PF cannot withstand force, *F*, with which the purple sphere is dragged upwards. **f** Dependence of the force a PF can withstand, as a function of the PF bending stiffness, expressed in two ways on the two *X*-axes.

Our recent cryoET study showed that curved tubulin PFs are features of MT ends during assembly and disassembly, both in cells and in vitro^25^. Modeling has suggested that the curved but flexible PFs straighten frequently by thermal fluctuations, providing a mechanism for MT elongation. However, PFs must be sufficiently rigid to explain the large forces generated during MT depolymerization^11,28,29^. Here, we ask whether these opposing constraints on PF properties can be harmonized into a single model for tubulin polymerization. We model a single PF by spheres connected via stretchable bonds (Fig. 1a). We assume that deviations from the PF’s naturally curved shape obey a simple Hookean law: *W* = *B* (*θ* − *θ*_0_)^2^/2, where *B* is the harmonic bending stiffness coefficient, *θ* and *θ*_0_ are current and equilibrium bending angles between adjacent tubulin subunits (Fig. 1b). *θ*_0_ is 0.2 rad, in agreement with measured curvatures of tubulin dimers^21–24^ and the shapes of PFs at the tips of growing and shortening MTs^25^. PF flexibility has previously been estimated in diverse ways, including the flexibility of intact MTs^30^, the force generated by depolymerizing MTs^11,28,29^, MT indentations by atomic force microscopy^31^, and variation in PF curvatures observed by cryoET^25^. Resulting estimates of PF persistence length range from 0.2 to 4.2 µm. These values correspond to harmonic bending stiffnesses of 14−300 kcal mol^−1^ rad^−2^ (Supplementary Fig. 1).

Given this span, we have simulated 2D Brownian motions of a single PF with a range of bending stiffnesses. For each, we counted the number of passages per second from curved to straight for the lowest tubulin dimer in the curved part of the PF. As shown in Fig. 1c, Brownian fluctuations drive PF straightenings at ~MHz rates over the whole range of PF bending stiffnesses. Longer PFs do experience higher drag, so the frequency of passages of from curved to straight decreases, but for the lowest tubulin dimer in the PF curve, it still exceeds 10^5^ Hz (Fig. 1d). Thus, the predicted frequency of PF fluctuations is significantly faster than the rate of arrival and loss of tubulin subunits from a PF tip (1−40 s^−1^ at 10 µM tubulin^32,33^). Therefore, PF bending stiffness does not limit MT growth rate in our mechanism of MT assembly.

To estimate the maximal force from a single bending PF as a function of bending stiffness, we have introduced a spherical object into the simulation (Fig. 1e). This represents a single subunit of the circular yeast kinetochore complex, Dam1^5,6^. The sphere is dragged toward the plus-end of the PF with a constant force parallel to the MT axis, exerting a load on the curved PF. The tendency of the PF to stay curved balances the load from the sphere. In these simulations the maximal force a PF can sustain varies linearly with bending stiffness (Fig. 1f). Optical tweezers experiments have demonstrated that a disassembling MT can develop up to 30 pN^11^, corresponding to ~2.3 pN per PF, assuming their contributions are independent and additive. If all PFs contribute their maximal force, the lowest PF bending stiffness that can generate 30 pN from 13 PFs, is ~35 kcal mol^−1^ rad^−2^ (Fig. 1e, f). However, if PFs are less efficient, the same force requires more rigid PFs. The highest PF bending stiffness in the literature corresponds to a stiffness coefficient of ~300 kcal mol^−1^ rad^−2^ in our model. We have therefore used an intermediate value, 174 kcal mol^−1^ rad^−2^, previously estimated from the energy of GTP hydrolysis^9,34^, as a default throughout this paper, unless stated otherwise.

### Lateral bond activation energy controls MT force generation

**Fig. 2:**
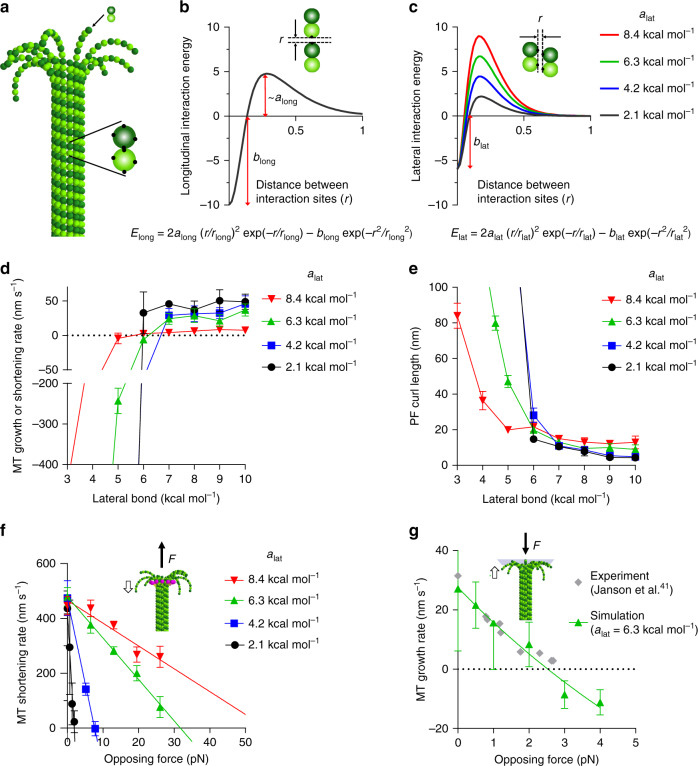
Role of lateral tubulin activation energy for MT dynamics and force generation. **a** Visualization of an MT, β- and α-tubulins depicted as in Fig. 1. Each tubulin monomer has two lateral and two longitudinal interaction sites (black dots). **b** Energy of longitudinal interaction between tubulin monomers as a function of distance between the interaction sites. Formula below describes the shape of longitudinal tubulin energy potentials. **c** A set of energy curves, describing lateral tubulin−tubulin interactions as a function of distance with different lateral activation energies (*a*_*lat*_). Strength of the lateral bond, *b*_*lat*_, equals 6 kcal mol^−1^ in this example. **d** Dependence of MT growth or shortening rate on the lateral bond strength, *b*_*lat*_, for different lateral barrier parameters, *a*_*lat*_. Numbers less than zero imply shortening. **e** Dependence of PF curl length on the strength of the lateral bond, *b*_*lat*_, graphed for several lateral activation energies, *a*_*lat*_. **f** Dependence of MT shortening rate on opposing force in simulations. The strength of the lateral bonds between the tubulins (*b*_*lat*_) was set to be weak, representing tubulins in the GDP state. Specifically, with each activation barrier height (*a*_*lat*_), *b*_*lat*_ was selected to enable shortening at ~400 nm s^−1^. See Source Data file for a full list of parameter values in all simulations. **g** Dependence of MT growth rate on opposing force in simulation and experiment. Tubulins were configured to have strong lateral bonds, *b*_*lat*_ = 8 kcal mol^−1^, to represent the GTP-state of tubulins. No GTP hydrolysis was allowed. Data points, describing simulation results in all graphs in this figure, represent mean ± s.d. based on 3–6 repeats of each simulation. Source data are provided as a Source Data file.

In an MT wall, the PFs are not independent; lateral interactions between them counteract their tendency to bend. To determine how this affects force production, we have simulated an MT with 13 laterally interacting PFs (Fig. 2a), by modifying an older model^34^ in two ways. First, in the new model, GTP- and GDP-tubulins have equal equilibrium bending angles *θ*_0_ = 0.2 rad between adjacent tubulin subunits^25^. Second, PF bending stiffness and lateral bonds between tubulins depend on the nucleotide state. Consistent with recent observations of MT tip shape^25^, each PF is confined to move in the plane that contains both the PF and the MT axis. The motion of each tubulin subunit can therefore be described by three planar coordinates: two for position and one for rotation. α- and β-subunits of the tubulin heterodimer are treated identically. Soluble GTP-bound α/β-tubulin dimers are not modeled explicitly; rather, they are added stochastically to the tips of PFs. The probability of adding a new α/β-tubulin dimer is equal for all PFs and is proportional to both the concentration of soluble tubulin and the tubulin association rate constant. In the MT lattice, tubulin subunits experience Brownian motion in the plane of PF bending. Tubulin−tubulin interactions are localized to four points positioned on the surface of each subunit: two for lateral interactions between monomers in adjacent PFs and two for longitudinal interactions along a PF (Fig. 2a). These interactions are described with energy functions that include both a potential well and an activation energy barrier (Fig. 2b, c). Although activation energy barriers are not commonly emphasized, they are likely a common characteristic of protein−protein interactions^35^. For tubulin, the barrier probably results from electrostatic repulsion between negative surface charges and the entropic penalty for eliminating solvent from protein’s surfaces.

Initially, we consider MTs made from tubulins in a single nucleotide binding state. When all other model parameters are fixed (Supplementary Table 1 shows values used), varying either the strength of lateral bonds (*b*_*lat*_ in Fig. 2d) or the PF bending stiffness (*B* in Supplementary Fig. 2a) changes the rate of MT assembly. The values of either parameter can define a transition from MT growth (positive) to shortening (negative); it is their balance that is key. Therefore, the GTP- and GDP-states of tubulin in the model can be given lateral bond strength and PF bending stiffness that promote either MT growth or shortening, with values that match the rates from experimental measurements. The lateral activation energy barrier (*a*_*lat*_) determines the steepness of the dependence of MT growth or shortening rates on both lateral bond strength (Fig. 2c, d) and PF bending stiffness (Supplementary Fig. 2a). Likewise, the lateral activation energy barrier controls the steepness of dependence of average PF curl length on those same parameters (Fig. 2e, Supplementary Fig. 2b). When the lateral activation barrier is small, the model is not robust in the sense that MT assembly and disassembly rates are overly sensitive to small changes in lateral bond strength or PF bending stiffness (blue curves in Fig. 2d and Supplementary Fig. 2a, respectively). Increasing the lateral activation barrier makes all these relationships more gradual and realistic.

To determine the magnitudes of forces that shortening model MTs can generate, we have introduced bonded spheres with flexible tubulin linkers to represent a Dam1 ring. Our approach follows that previously proposed^9^ with modifications (see “Methods”). When a constant force applied to each subunit of the Dam1 ring is strong enough to overcome the binding energy between Dam1 linkers and tubulin, the ring can be dragged toward the plus-end of the depolymerizing MT. With a low activation barrier (e.g. *a*_*lat*_ = 2.1 kcal mol^−1^), the theoretical MTs, which shorten under zero load at ~400 nm s^−1^, are stalled by an opposing force of ~2.5 pN (Fig. 2f, Supplementary Movie 1), regardless of PF bending stiffness (Supplementary Fig. 2c). The presence of a high activation energy barrier enables the generation of large forces by disassembling MTs (Fig. 2f). The stall force in the simulations with *a*_*lat*_ = 6.3 kcal mol^−1^ matches experimental measurements^11^.

Thus, the activation energy barrier is a key, previously underappreciated parameter of lateral tubulin−tubulin interaction; it determines MT behavior under force and makes MT dynamics robust to small changes in lateral bond strength and PF stiffness.

### Our model also describes pushing by growing MTs

MTs have long been known to develop pushing forces during polymerization^36^. These forces influence the internal organization of cells^37–40^. A push of 2.4–4 pN can be generated in vitro with 20 µM tubulin^36,41,42^. To examine the generation of pushing forces, we have simulated MT polymerization against an obstacle that exerts a constant resistance (Fig. 2g, Supplementary Movie 2). The stall forces for simulated growing MTs at a given free tubulin concentration are determined mainly by the balance between the lateral bond strength and PF bending stiffness (Supplementary Fig. 2b). With a high activation energy barrier and intermediate PF bending stiffness, the model provides a good match to experimental data (Fig. 2g). The model also predicts MT depolymerization under high loads (negative values in Fig. 2g), implying that high loads could induce a polymerization catastrophe, consistent with previous reports^43^.

### Modeling suggests two mechanisms for dynamic instability

**Fig. 3:**
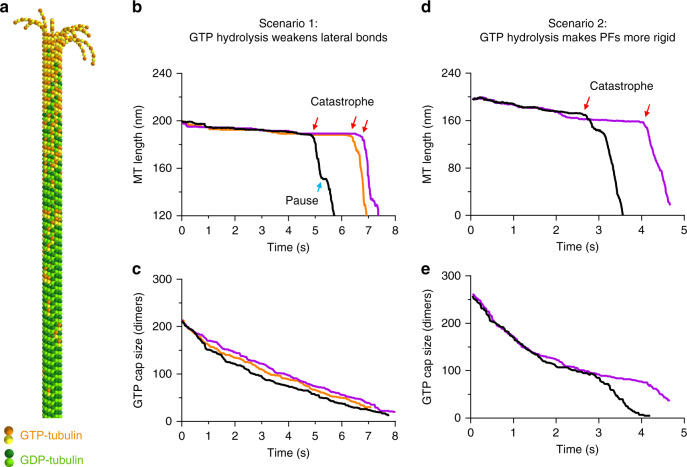
Possible mechanisms of MT lattice destabilization by GTP hydrolysis. **a** Visualization of the starting configuration of an MT in the tubulin dilution simulations. GDP-bound tubulin dimers are shown with light and dark green spheres. GTP-bound dimers are shown with yellow and orange spheres. **b** Dependence of MT length on time in three independent repeats of the tubulin dilution simulation: PF bending stiffness is identical for GTP- and GDP-bound PFs and equal to 174 kcal mol^−1^ rad^−2^; lateral bond strength, *b*_*lat*_ equals 8 and 4.7 kcal mol^−1^ for GTP- and GDP-bound tubulins, respectively. Blue arrow indicates a brief pause of rapid GDP-MT lattice disassembly at a random GTP-tubulin remnant, which happened occasionally in some simulation runs (also see Supplementary Movie 3). **c** Dependence of the total number of remaining GTP-tubulin subunits on time in three independent repeats of tubulin dilution simulation from panel **b**. **d** Dependence of MT length on time in two independent repeats of tubulin dilution simulation: the lateral bond strength is set to 4.7 kcal mol^−1^, regardless of the bound nucleotide; PF bending stiffnesses are 78 and 174 kcal mol^−1^ rad^−2^ for GTP- and GDP-bound PFs, respectively. **e** Dependence of the total number of remaining GTP-tubulin subunits on time in two independent repeats of tubulin dilution simulation from panel **d**.

Analysis of model MT behavior suggests that a switch from assembly to disassembly could be driven by changes in either lateral bond strength or PF bending stiffness, both of which are plausible results of GTP hydrolysis. These changes can be realized in several ways. If PF bending stiffness is fixed, simulated MTs will assemble when lateral bonds are strong, but will disassemble if the bonds weaken (Fig. 2d). Alternatively, when the lateral bond strength is fixed, MTs will assemble if the PFs are flexible and disassemble if they become stiffer (Supplementary Fig. 2a). To illustrate how such changes could induce a catastrophe (a stochastic transition from growth to shortening), we implemented random GTP hydrolysis and made hydrolysis lead either to a decrease in the strength of lateral interactions or an increase in PF bending stiffness. In the presence of 5–20 µM soluble tubulin, MT catastrophes are expected only once per hundreds of seconds^44–46^, an occurrence too rare to model in a reasonable computation time. We therefore simulated a dilution of soluble tubulin to zero concentration, a condition in which MT catastrophes happen within a few seconds^1,47^. Initially, MTs were given a GTP cap of ~110 nm, corresponding to 200–250 tubulin dimers, consistent with experimental estimates^1^. The GTP-tubulins were distributed exponentially with the highest concentration at the flared plus-end (Fig. 3a). The hydrolysis rate was 0.24 s^−1^, as reported^1,48^. We then introduced two parametrizations for GTP- and GDP-tubulins: (1) different nucleotide-defined lateral bond strength (Fig. 3b, c), or (2) different PF bending stiffness (Fig. 3d, e). Meanwhile, the lateral activation energy barrier was kept constant. With both parametrizations, MTs disassembled in two distinct phases: first, the GTP cap gradually disappeared, then the MT showed rapid depolymerization, a characteristic of GDP-lattice disassembly (Fig. 3b–e, Supplementary Movies 3, 4). We suggest that in the presence of soluble tubulin, MT catastrophes occur in a similar fashion, although it takes more time than in dilution experiments for the GTP cap to disappear. Our current model cannot distinguish between the two proposed effects of GTP hydrolysis, suggesting that either or both could pertain.

### Tubulin dynamics as a function of tubulin concentration

**Fig. 4:**
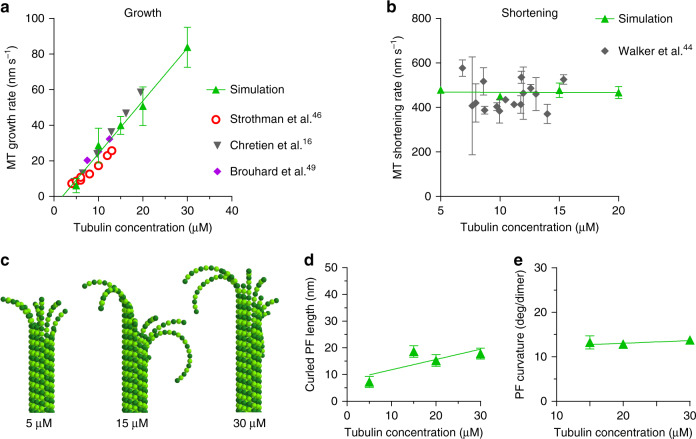
Dependence of MT assembly and disassembly on free tubulin concentration. **a** MT growth rate vs. tubulin concentration in experiments and in the simulation. GTP hydrolysis constant was set to zero. GTP-tubulins were assumed to have strong lateral bonds: *b*_*lat*_ = 8 kcal mol^−1^. Full list of model parameters is given in Supplementary Table 1 and Source Data file. **b** Dependence of MT shortening rates on free GTP-tubulin concentration in published experiments and in simulation. At the onset of the simulation MTs were composed entirely of GDP-tubulins. They depolymerized in spite of transient additions of GTP-tubulins at the tips of curved PFs. Full list of model parameters is given in Supplementary Table 1 and Source Data file. **c** Shapes of growing GTP-MT tips in simulations at three tubulin concentrations. **d**, **e** GTP-PF curl lengths and average PF curl curvatures as functions of tubulin concentration in simulations. Data points, describing simulation results in all graphs in this figure, represent mean ± s.d. based on 3–6 repeats of each simulation. Source data are provided as a Source Data file.

To investigate the mechanism of MT assembly, we simulated MT growth from nonhydrolyzable GTP-tubulins. Despite the presence of curved PFs at the tip, model MT growth rate is a linear function of tubulin concentration, in agreement with experimental data (Fig. 4a)^16,46,49^. The slope of the line is primarily determined by the tubulin association rate constant. For a given value of this constant, the critical tubulin concentration is dictated by the strengths of both longitudinal and lateral bonds. Only relatively slow association rate constants, e.g., 0.6 s^−1^ µM^−1^ per PF, can simultaneously describe the experimentally observed dependence of MT growth rate on tubulin concentration and the average lengths of PF curls at the growing MT ends^25^. This on-rate is similar to the recent, direct measurement for yeast tubulin^50^, though considerably lower than the values theoretically inferred by Gardner et al.^32^. The rate of MT shortening is insensitive to GTP-tubulin concentration, consistent with experimental data (Fig. 4b)^44^.

### MT end structure as a function of tubulin concentration

The linear dependence of MT growth rate on tubulin concentration in the model with curved PFs at the tips is less intuitive than in models with straight MT tips. One might expect that higher concentrations of soluble tubulin would increase the rate of elongation at each PF, while the rate of lateral bond formation should not be affected, making the curled PFs longer. However, due to the high rate of thermally driven PF fluctuations, lateral bond formation and breakage in our model are significantly more frequent than the arrival/loss of new subunits at the PF tips. PFs zip and unzip repeatedly at the growing MT tip, reaching a dynamic steady state between any consecutive events of tubulin arrival/loss. With a high lateral bond activation energy, the frequency of spontaneous straightening becomes lower, but even when *a*_*lat*_ = 6.3 kcal mol^−1^, the rate of spontaneous PF straightening substantially exceeds the frequency of tubulin arrivals in the physiological range of tubulin concentrations. Therefore, MT growth is limited by the rate of new tubulin attachments to the ends of protofilaments, not by the rate of lateral bond closure. An interesting prediction is therefore that the lengths and curvatures of curled PFs during MT growth should be insensitive to the concentration of soluble tubulin (Fig. 4c–e).

### Experimental tests of model predictions

**Fig. 5:**
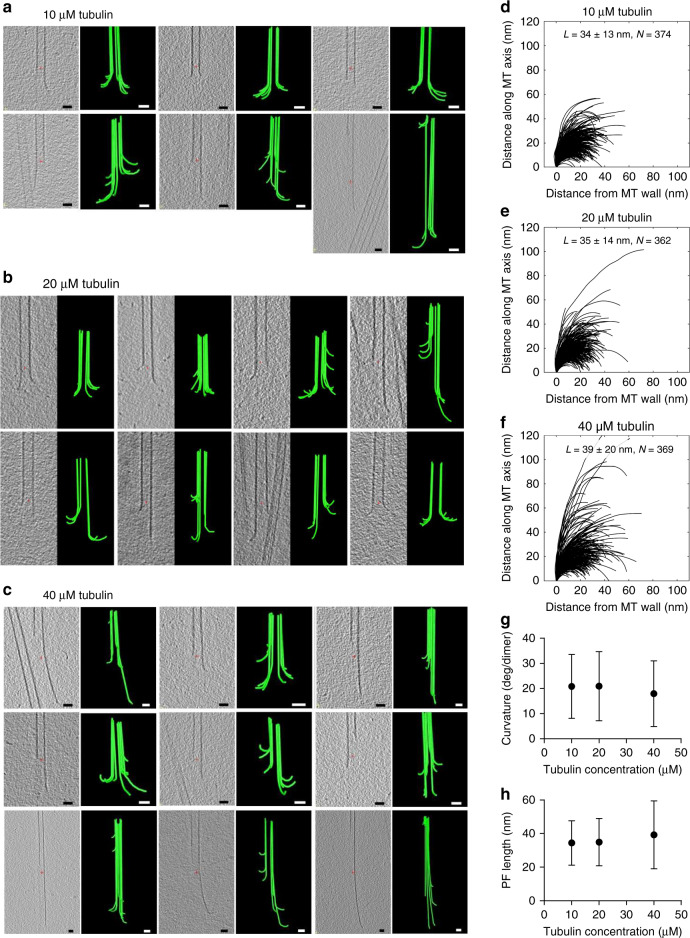
Quantitative analysis of PF tip shapes on MTs grown at three tubulin concentrations. **a**–**c** Tomographic slices and models of representative MTs elongating from axonemal doublet MTs at three concentrations of free tubulin. Red crosses mark the origins of the coordinate systems used. Bars, 25 nm. **d**–**f** LOESS-smoothed traces of PFs from 136 growing MTs (49 MTs at 10 μM, 37 MTs at 20 μM, and 50 MTs at 40 μM). Ns numbers of PFs traced. Raw tracing coordinates are provided as a Source Data file. **g** Average curvatures of PFs as a function of free tubulin concentration. Means ± s.d. **h** Average curled PF lengths as a function of free tubulin concentration. Means ± s.d. Source data, describing PF curvatures and lengths, are provided as a Source Data file.

To test this prediction, we used cryoET with rotational sampling to examine the tips of MTs elongating at different rates. We grew MTs from axonemes in vitro at three concentrations of soluble tubulin: 10, 20, and 40 μM. The samples were prepared and analyzed as previously described^25^. Typical slices through tomographic reconstructions of MT tips and graphic models drawn on the tip reconstructions are shown in Fig. 5a–c. Figure 5d–f displays hand-drawn traces on all PFs identified at every MT tip from each growth condition. Quantifications of PF lengths and average curvatures show no significant dependence on tubulin concentration, in agreement with prediction (Fig. 5g, h, Supplementary Table 3).

**Fig. 6:**
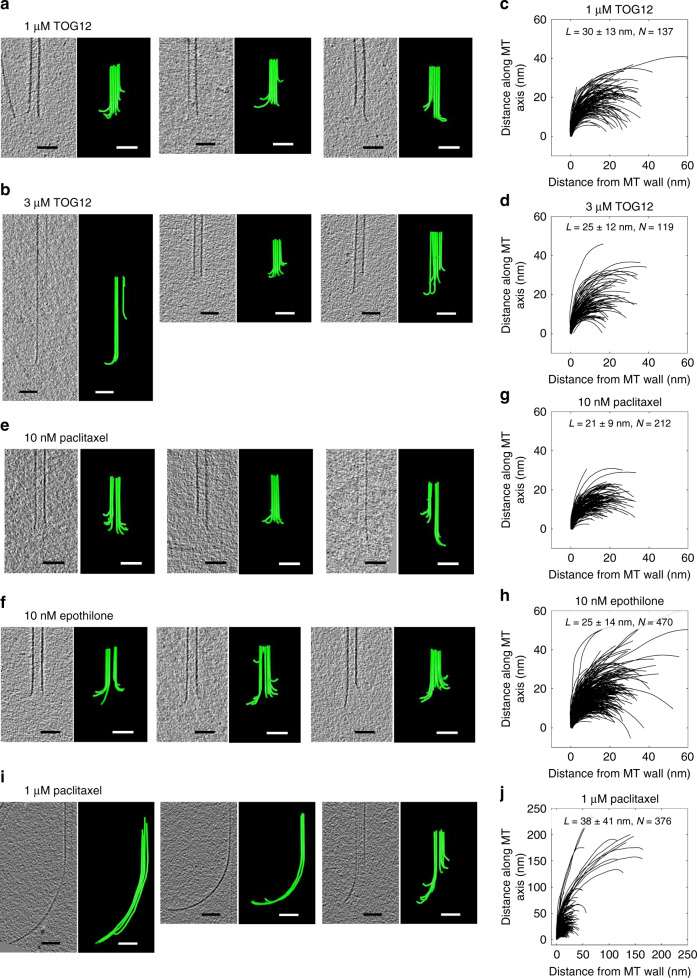
Effects of an MT polymerase and assembly-inhibiting drugs on PF tips. **a**, **b** Tomographic slices and models of representative MTs elongating from axonemal doublet MTs in presence of 20 μM free tubulin and two concentrations of TOG12 protein. Bars, 50 nm. **c**, **d** LOESS-smoothed traces of PFs from 16 MTs growing in the presence of 1 μM TOG12 protein and 12 MTs growing in the presence of 3 μM TOG12 protein, respectively. *N* numbers of PFs traced. **e**, **f** Tomographic slices and models of representative MTs elongating from axonemal doublet MTs in the presence of 20 μM free tubulin and 10 nM paclitaxel or 10 nM epothilone. **g**, **h** LOESS-smoothed traces of PFs from 22 MTs growing in the presence of 10 nM paclitaxel and 47 MTs growing in the presence of 10 nM epothilone, respectively. *N* numbers of PFs traced. **i** Tomographic slices and models of representative MTs elongating from axonemal doublet MTs in the presence of 20 μM free tubulin and 1 μM paclitaxel. **j** LOESS-smoothed traces of PFs from 36 MTs growing in the presence of 1 μM paclitaxel. *N* numbers of PFs traced. See Supplementary Table 3 for mean curvatures and PF lengths in each condition. Raw tracing coordinates for all conditions are provided as a Source Data file.

In a second set of experiments, we altered the rate of MT elongation at a constant concentration of soluble tubulin by adding different concentrations of a TOG protein to the polymerization solution. We used TOG12, an N-terminal fragment of the potent MT polymerase, XMAP215. This polymerase lacks a segment that binds the MT wall, but at a concentration similar to the tubulin dimers it is an effective polymerase^51^. In our hands, TOG12 at 1 and 3 μM increased the elongation rates of MTs growing from axonemes in 20 μM tubulin by ~2- and ~4-fold, respectively. PF lengths and curvatures were similar to those on MTs growing without this catalyst, as predicted (Fig. 6a–d, Supplementary Table 3).

Some MT-targeting drugs at substoichiometric concentrations slow MT growth^52^, so we asked if 10 nM epothilone or paclitaxel would alter the shapes of growing MT tips. The lengths and curvatures of the resulting PF curls were unaffected by these treatments (Fig. 6e–h, Supplementary Table 3). At higher concentrations of paclitaxel, however, the shape of growing MT ends changed significantly (Fig. 6i). In 1 μM paclitaxel, about 5% of the MTs formed long clusters of a few PFs with a gentler curvature than individual PFs. This observation cannot be interpreted by our model, because it does not allow deviation of PFs from their individual radial planes. However, this pronounced change of MT tip shape in high paclitaxel demonstrates the ability of our imaging procedure to detect structural variations at MT ends, serving as an intrinsic control.

### MT end raggedness is a function of polymerization conditions

**Fig. 7:**
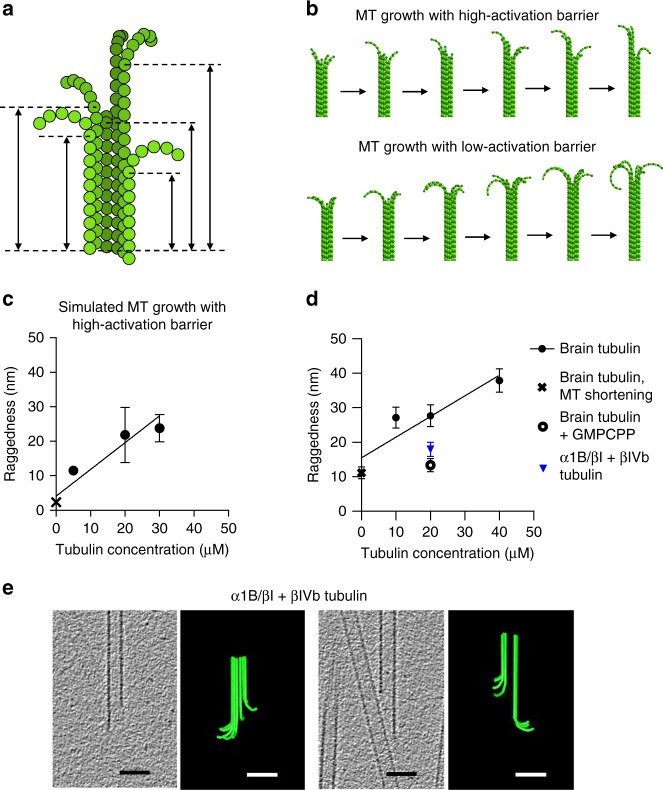
Raggedness of MT tips grown in different conditions. **a** Schematic explaining the MT end raggedness parameter. Dashed lines mark the origins of PF curls. Double-ended arrows show distance from a fixed arbitrary point to the start of each PF curl. Raggedness is defined as the standard deviation of positions of PF curve starts. **b** Series of snapshots, demonstrating growing GTP-MT end shapes in simulations with high and low lateral activation energies for lateral bonding potentials. More ragged ends tend to form when the activation barrier is high. **c** Dependence of model GTP-MT end raggedness on tubulin concentration. Data points represent mean ± s.d. from thee repeats of each simulation; source data are provided as a Source Data file. **d** Experimental data on MT raggedness under various experimental conditions. Black points show dependence of MT end raggedness on tubulin concentration. Black line is a linear fit to these data. Zero tubulin concentration (cross) corresponds to isothermal dilution experiments. Open circle shows data for MT growth in the presence of GMPCPP, blue triangle is growth in α1B/βI+βIVb tubulin. All data points show mean ± s.e.m. based on measurements for 15–97 MTs; source data are provided as a Source Data file. All data points show mean ± s.e.m. **e** Tomographic slices and models of representative MTs elongating from axonemal doublet MTs in the presence of 20 μM free α1B/βI+βIVb tubulin.

Another prediction of the model relates to the raggedness of MT ends. We define the raggedness of each MT as the standard deviation of the axial positions at which PF begins to curl (Fig. 7a). This parameter is reminiscent of a previous measure of MT tip taper, calculated as the standard deviation of PF lengths^32^. The difference between these measures is that the resolution of cryoET allows each PF to be treated separately. The model predicts that the extent of raggedness grows over time, so MTs develop shapes in which some PFs lag behind others (upper row of Fig. 7b, Supplementary Movie 5). The extent of model MT end raggedness depends on tubulin concentration (Fig. 7c), and the raggedness of depolymerizing MTs is predicted to be low (Fig. 7c, cross). Note that in the simulations with a small lateral activation energy barrier (*a*_*lat*_ ≤ 2.1 kcal mol^−1^), the predicted raggedness of assembling MTs is low and only weakly dependent on tubulin concentration (lower row of Fig. 7b, Supplementary Fig. 3a).

We tested these predictions by examining growing and shortening MT ends with cryoET. MT growth was assured by using concentrations of tubulin well above the critical concentration for assembly, by using axonemes to nucleate polymerization, and by sampling soon after growth initiation. Moreover, observations over time confirmed that our polymers were elongating^25^. Shortening polymers were obtained by a 25-fold isothermal dilution with tubulin-free polymerization buffer^25^. Consistent with simulations using high lateral activation barriers, the mean raggedness of MT tips was substantially higher for growing MTs than for depolymerizing MTs (Fig. 7d, Supplementary Fig. 3b), but the experimentally observed raggedness was higher than predicted by the model. One explanation for this difference is the short time span of our simulations (a few seconds), which may not faithfully represent MT growth over minutes. Another factor may be that the MTs we studied by cryoET were polymerized from brain tubulin, a mixture of tubulin isotypes and post-translational modifications^53^. Such variation in protein structure could lead to a diversity in polymerization behavior. To minimize this heterogeneity, we repeated the observations, using tubulin with reduced complexity, which contains primarily one α- and two β-tubulin isoforms (α1B/βI + βIVb) and no detectable post-translational modifications^54^ (Supplementary Fig. 3c, d). The raggedness of these MT tips was lower than that observed with brain tubulin (Fig. 7d, e), matching the model’s predictions better than brain tubulin. More blunt ends were also observed previously with this tubulin species^54^, but that study did not include a tomographic analysis of individual PFs. Intriguingly, the ends of MTs elongating with brain tubulin in the presence of GMPCPP were fairly even (Fig. 7d, open circle). This difference might reflect a fundamental difference between GMPCPP- and GTP-tubulins, like a lower activation barrier for lateral interactions. Such a change would also explain enhanced MT nucleation with this nucleotide, but we cannot exclude the possibility that decreased tip raggedness came from a lower tubulin concentration in the GMPCPP experiments. The nominal tubulin concentration was 20 µM, but some loss of free tubulin was possible due to spontaneous MT nucleation, and/or sticking of protein to the concentration filter used during nucleotide exchange (see “Methods”).

### Poor correlation between the shapes of neighboring PFs

To seek further comparison between theory and experiment, we asked if there was any inter-dependence in the lengths or curvatures of PFs as a result of their relative positions at an MT tip. In our simulations, the lengths of PF curls showed no correlation, regardless of position. For example, during MT growth the Spearman correlation between the lengths of adjacent PFs was: *r*_s_ = −0.02, with (two-tailed) *p* value > 0.05; for the average curvatures of PF curls: *r*_s_ = −0.01, (two-tailed) *p* value > 0.05. To test this prediction, we collected information about the shapes of individual PF traces whose relative positions were known from cryoET. Spearman correlations between either the lengths or curvatures of adjacent PF curls during MT growth at 20 µM tubulin were low (*r*_s_ = 0.32, with (two-tailed) *p* value < 0.01 and *r*_s_ = 0.16, with (two-tailed) *p* value < 0.01, respectively), supporting the idea that PF curls are independent.

### MT growth with curved PFs is accelerated by assisting forces

**Fig. 8:**
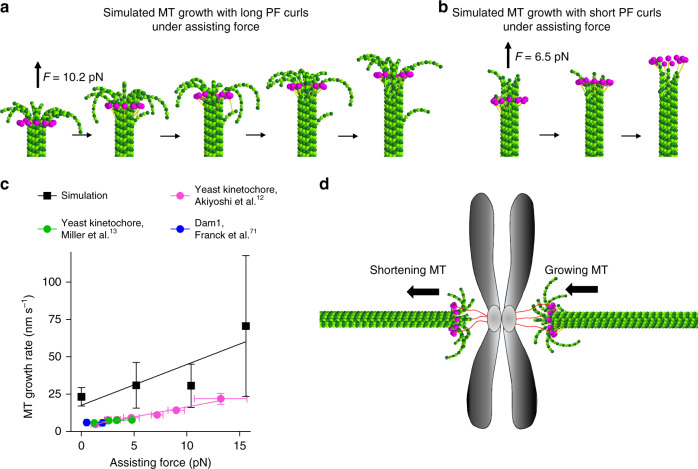
MT growth with flared PFs can sustain assisting load and be accelerated by it. **a** Snapshots from a simulation of GTP-MT growth with long PF curls in the presence of a Dam1 ring. GTP hydrolysis is turned off. **b** Snapshots from a simulation of GTP-MT growth with ring and short PF curls. GTP hydrolysis is turned off. **c** Dependence of GTP-MT growth on assisting force in the model (10 µM tubulin, 37 °C) and experimental data (15 µM tubulin, 23 °C). The difference in MT growth rates between the simulation and experiments is likely due to the distinct temperature conditions. Simulation data points represent mean ± s.e.m. based on three repeats of each simulation; source data are provided as a Source Data file. **d** Schematic illustrating the proposed principle of synchronization of growing and shortening MTs during chromosome oscillations in mitosis.

The similarity of growing and shortening MT ends hints that their kinetochore coupling mechanisms could be similar. We used simulations to ask if the flared morphology of growing ends could enhance coupling with Dam1 ring motions under force^12,13^. We modeled a constant, plus-end directed force on a Dam1 ring. After arriving at the MT tip, the ring was stopped by the flared PFs, enabling tip tracking with the growing end (Fig. 8a, Supplementary Movie 6). The ability of the growing tip to retain the ring depended on the length of the curled PFs (Fig. 8b, Supplementary Movie 7). However, if PFs were straight, like those previously considered^34^, no binding affinity between tubulins and the ring’s linker could enable the tracking under assisting force. This demonstrates that the flared morphology of a growing end is beneficial for growing MT end tracking. Interestingly, assisting forces accelerated MT assembly rate in simulations (Fig. 8c). Increased speed is achieved through promoting lateral bond closure. In the absence of catalysts, i.e. when the association rate constant equaled 0.64 s^−1^ µM^−1^ per PF, the maximal extent of MT assembly acceleration by force is ~2-fold. With a higher association rate constant, however, the acceleration factor increased (Supplementary Fig. 4). The ability of MTs to grow faster under assisting forces has been documented in studies that used yeast kinetochores and purified XMAP215 polymerase^12–14^. These observations have not been explained in the framework of previous models, and they have remained a puzzle, as recently discussed^55^; our model resolves this problem.

## Discussion

Here we report our examination of a Brownian dynamics model for MT assembly and disassembly that accommodates the curved shape of GTP-tubulins^21–24^ and our finding of curved PFs at the tips of growing MTs^25^. Most previous models have assumed straight PFs at growing MT ends, so tubulin dimer attachment or detachment involved simultaneous formation or breakage of lateral and longitudinal bonds^34,56–60^. An exception is the model of Margolin et al.^61^, which considered lateral cracks between PFs without breakage of longitudinal bonds. Those models have described MT ends as blunt or ragged. To the best of our knowledge, formation of sheet-like structures at growing MT ends has not been predicted by any of the published models for MT dynamic instability.

Our model considers structural features of MT ends, observations of MT dynamics, and it accounts for MT force generation, a source of information that provides constraints and important insights into tubulin−tubulin interactions. Most previous models did not take force generation data into account, and therefore failed to describe them (see Supplementary Table 4). The only other attempt to consider both dynamic instability and force generation within a single model, of which we are aware, is the study by Schek et al.^62^. They applied a Monte-Carlo model to examine MT assembly under opposing loads, but did not describe force production by MT disassembly. Forces developed by shortening MTs were previously considered with a Monte-Carlo Metropolis model of MT depolymerization or inferred from a static molecular-mechanical model by our group, but without consideration of dynamic instability^7,9,63,64^. This gap motivated us to integrate MT dynamics, force development, and MT end structure into one quantitative framework. We find that the key, previously underappreciated parameter controlling force generation is the high and steep activation energy barrier in the lateral tubulin−tubulin interaction potential. When activation energy is high, both shortening and growing MTs can develop large pulling forces, consistent with experimental data^10–13,28,29^. Although part of this barrier may result from electrostatic repulsion between tubulins and from the entropic penalty for eliminating solvent from the relevant protein surfaces, it may also depend on degrees of freedom not present in our current model, given its simplicity. For example, our molecular dynamics study indicates that PFs twist slightly around their axes when they splay out from the MT lattice^65^. This implies that simple reversal of the bending motion may not suffice to re-form the lateral bond. (Counter-clockwise twisting is required to return to the initial state, diminishing the frequency of transition from curved to laterally bound states, which is represented by the high energy barrier between those states.)

In the model of MT assembly with straight GTP-PFs at the tip, MT growth rate can be controlled only through the changes in tubulin on- and off-rates. This pathway is likely regulated by TOG polymerases, which have been suggested to increase the effective tubulin association rate constant^49,66,67^. Our model opens an additional possibility for accelerating MT growth through favoring lateral bond formation between GTP-tubulin PFs where they meet in the MT wall. Proteins like EB1 are well positioned to do this job^68^; they might use this pathway to accelerate MT growth. Under normal conditions for MT assembly in vitro, EB-proteins accelerate MT growth by only ~1.5-fold^69^. In our interpretation, this increase is small because MT growth under those conditions is limited by the tubulin on-rate. In contrast, XMAP215 in vitro accelerates MT growth by as much as 5-fold^49^. We hypothesize, that at high XMAP215 concentrations, the tubulin on-rate becomes comparable with the frequency of lateral bond closure. This increases the sensitivity of MT growth rate to modulation of lateral bond formation by EB1, hence the synergetic effect of XMAP215 and EB1^69^. Reported synergy between XMAP215 and paclitaxel might have a similar origin. At 1 μM paclitaxel, we find that PFs at growing MT ends form long clusters with gentler curvature. In all other conditions tried, PFs stay in their radial planes, based on cryoET, suggesting that PF rigidity for out-of-plane movements is very high^25^. However, the presence of paclitaxel may alter this situation, so a more careful analysis with a full 3D model of MT assembly should be carried out.

The curved morphology of PFs at growing and shrinking MT tips suggests consistent mechanisms of kinetochore coupling to dynamic MT tips. Thus, kinetochores would not have to adapt to structural changes at MT tips during their transition from growth to shortening and back, as anticipated in previous views of growing MT ends. Curved PFs at growing MT tips explain the observation of flared PFs at both leading and trailing kinetochores of oscillating chromosomes^70^. In contrast to blunt-ended or sheet-like MT growth models, our model allows regulation of the assembly rate by an assisting force. This is consistent with the observed increase in polymerization velocity upon application of an assisting force^12–14,71^. In cytoplasm, where assembly catalysts are present, MT growth could be accelerated several-fold by application of a plus-end directed force. Such sensitivity of growth rate to assisting force provides an attractive way to synchronize the velocities of MT growth and shortening at the trailing and leading kinetochores of oscillating chromosomes during metaphase (Fig. 8d), shedding light on this long-standing problem.

## Methods

### Simulation of a single PF

An isolated tubulin PF was represented by a set of 16 spheres (8 tubulin dimers). A position restraint was applied to its bottom (minus-end proximal) dimer. All other spheres were treated identically and they were described with the two coordinates of the center of mass {*x*, *y*} and a rotation angle *τ*. The spheres, which form a single dimer, were connected with Hookean springs of stiffness *k* = 310 kcal mol^−1^ nm^−2^:

1 $$U(r) = \frac{k}{2}r^2,$$

where *r* is the distance between the longitudinal interaction points on the spheres, *U* is the energy of longitudinal bond stretch. Additionally, a bending potential was applied to make the equilibrium shape of the PF curved:

2 $$W(\theta ) = \frac{B}{2}\left( {\theta - \theta _0} \right)^2,$$

where *W(θ)* is the energy of tubulin rotational strain, *B* is the flexural rigidity coefficient, *θ* is the bending angle calculated as the difference between the rotation angles *τ* of a given tubulin monomer and its adjacent minus-end proximal neighbor, *θ*_0_ the equilibrium bending angle.

Motions of the spheres in each PF were described with Langevin equations, which were numerically solved using the Ermak and McCammon algorithm^72^. To measure the frequency of PF straightening, we simulated Brownian dynamics of 100 PFs for 0.1 s and measured the average frequency of the passages from curved to straight states of the lowest (minus-end proximal) nonrestrained tubulin monomer. The state of the tubulin was considered “curved” when its bending angle exceeded 0.15 rad, and “straight” when the bending angle was less than 0.05 rad.

To examine maximal forces that a single curved PF in the absence of lateral neighbors can develop, we carried out simulations of an isolated PF interacting with a sphere, dragged by a constant external force. Total energy of the PF depended on three terms: longitudinal stretch energy (Eq. 1), bending energy *W* (Eq. 2), and the energy of interaction with the sphere, described as follows:

3 $$\rho \left( d \right) = \left\{ {\begin{array}{*{20}{l}} {k_{{\it{{rep}}}}\left( {R + r - d} \right)^2,} \hfill & {{\mathrm{{if}}}\,d \,< \,R + r} \hfill \\ {0,} \hfill & {{\mathrm{{if}}}\,d \,\ge \,R + r} \hfill \end{array}}, \right.$$

where *k*_*rep*_ *=* 100 kcal  mol^−1^ nm^−2^ is the stiffness of tubulin-Dam1 subunits repulsion, restricting their penetration into each other, *R* *=* 3 nm and *r* *=* 2 nm are radii of Dam1 and tubulin subunits, respectively, *d* is the distance between their centers.

The Dam1 subunit was constrained to move in only the *z* direction parallel to the MT axis and located *x* = 7 nm from the axis of a straight PF. The total energy of the Dam1 subunit contained two terms: potential energy of the constant external force: *w*(*z*) = −*Fz*, and the energy of repulsion from tubulin subunits, described with Eq. (3). To find the maximal force, which a single PF could exert against a Dam1 subunit, dragged with a constant external load, we solved the force balance equation by applying formula (9) with temperature set to zero, which is mathematically similar to using the gradient descent iterative minimization algorithm.

Model parameters and their values, describing tubulin interactions with Dam1 subunits, or with an obstacle (see below in “Methods”) are enlisted in Supplementary Table 2.

To establish the relationship between the dynamic persistence length of PFs and the harmonic PF bending stiffness used in this model, we applied a script previously developed to measure the dynamic persistence length of PFs examined with cryoET. Briefly, the dynamic persistence length can be defined as the distance along the filament, at which the correlation between angular deflections of the filament from its equilibrium shape decays by a factor of *e*, due to thermal fluctuations^73^. This definition can be expressed with the following formula:

4 $$\left\langle {\vec \tau \left( 0 \right)\vec \tau \left( s \right)} \right\rangle = \left\langle {{\mathrm{cos}}\left( {\delta (s)} \right.} \right\rangle = e^{ - \frac{s}{{\xi _{\mathrm{d}}}}},$$

where $\vec \tau$(s) is tangent vector at position *s* along the filament, *δ*(*s*) is the filament’s position-dependent angular deflection from the average curved shape, and *ξ*_d_ is the dynamic persistence length.

### Computational model of MT

Our algorithm for modeling a full MT end, containing 13 PFs, contained two computational layers: a slow, kinetic Monte-Carlo layer, realizing rare stochastic tubulin additions to the PF tips and GTP hydrolysis; and a fast layer, implementing Brownian dynamics of tubulin dimers inside the MT lattice. The events of the slow layer occurred with *t*_*kin*_ = 13 ms time step. The probability of adding a new tubulin dimer was equal for the tips of all PFs and depended only on the concentration of soluble tubulin, *c*_*tub*_, and the tubulin association rate constant, *k*_*on*_:

5 $$P = k_{{\it{{on}}}}c_{{\it{{tub}}}}t_{{\it{{kin}}}}.$$

Random GTP hydrolysis could occur with equal rate constant *k*_*hydr*_ in any of the GTP-tubulins, leading to either a change of the lateral bond strength (*b*_*lat*_) or PF bending stiffness (*B*).

Longitudinal bonds between tubulin subunits within a dimer were modeled as stretchable Hookean springs of stiffness *k* (Eq. 1).

Inter-dimer longitudinal and lateral bonds were breakable and described by an energy potential with an activation barrier:

6 $$E_{{\it{{long}}}}(r) = 2a_{{\it{{long}}}}\left( {\frac{r}{{r_{{\it{{long}}}}}}} \right)^2\exp \left( { - \frac{r}{{r_{{\it{{long}}}}}}} \right) - b_{{\it{{long}}}}\exp \left( { - \frac{{r^2}}{{r_{{\it{{long}}}}^2}}} \right),$$

7 $$E_{{\it{{lat}}}}(r) = 2a_{{\it{{lat}}}}\left( {\frac{r}{{r_{{\it{{lat}}}}}}} \right)^2\,{\mathrm{exp}}\left( { - \frac{r}{{r_{{\it{{lat}}}}}}} \right) - b_{{\it{{lat}}}}\,{\mathrm{exp}}\left( { - \frac{{r^2}}{{r_{{\it{{lat}}}}^2}}}. \right)$$

Here *E*_*long*_(*r*) is the energy of inter-dimer longitudinal interaction, *E*_*lat*_(*r*) is the energy of lateral interaction, *r* is the distance between a pair of interaction points, *r*_*long*_ and *r*_*lat*_ are parameters, determining the widths of the bonds and the steepness of the activation energies, *a*_*long*_ and *a*_*lat*_ are parameters, characterizing the height of activation barriers, *b*_*long*_ and *b*_*lat*_ are the depths of the potential wells for longitudinal and lateral bonds, respectively.

The orientation of adjacent tubulin monomers relative to one another within a PF was described by a quadratic energy potential (Eq. 2).

Thus, the total energy of all subunits in the MT was

8 $$U_{{\it{{total}}}} = \mathop {\sum }\limits_{n = 1}^{13} \mathop {\sum }\limits_{k = 1}^{K_n} \left( {E_{{\it{{lat}}}}^{k,n} + E_{{\it{{long}}}}^{k,n} + U^{k,n} + W^{k,n}} \right).$$

Here *n* is the PF number, *k* is the number of the monomer in the *n*th PF, *K*_*n*_ is the total number of tubulin subunits in *n*th PF.

The coordinates of the all monomers of the system at the *i*th computation cycle were obtained by solving Langevin equations of motions^72^:

9 $$\left\{ \begin{array}{l}q_{k,n}^i = q_{k,n}^{i - 1} - \frac{{{\mathrm{{d}}}t}}{{\gamma _q}} \cdot \frac{{\partial U_{{\it{{total}}}}}}{{\partial q_{k,n}^i}} + \sqrt {2k_{\it{{B}}}T\frac{{{\mathrm{{d}}}t}}{{\gamma _q}}} \cdot N(0,1)\\ \tau _{k,n}^i = \tau _{k,n}^{i - 1} - \frac{{{\mathrm{{d}}}t}}{{\gamma _\tau }} \cdot \frac{{\partial U_{{\it{{total}}}}}}{{\partial \tau _{k,n}^i}} + \sqrt {2k_{\it{{B}}}T\frac{{{\mathrm{{d}}}t}}{{\gamma _\tau }}} \cdot N(0,1)\end{array} \right.$$

$$\gamma _q = 6\pi r\eta$$

$$\gamma _\tau = 8\pi r^3\eta$$

where *q* = {x, z} are the positions of the center of each tubulin subunit, *τ* is the tubulin subunit rotation angle, d*t* is the computation time step; *U*_*total*_ is given by Eq. (8); *k*_B_ is the Boltzmann constant; *T* is temperature; *N*(0,1) is a random number from a normal distribution, generated using the Mersenne twister algorithm^74^; *γ*_*q*_ and *γ*_*τ*_ are translational and rotational viscous drag coefficients, respectively, calculated for a sphere with radius *r* *=* *2* nm.

Any tubulin oligomer that separated from its PF, due to a Brownian fluctuation, far enough that its energy of longitudinal interaction with the PF dropped below 0.1 *k*_*B*_*T*, was considered “detached” and was removed from the simulation.

The computational algorithm was realized in C++ and run on HPC computing resources at Lomonosov Moscow State University. Scripts for visualization and processing results were written in Python 2.7.

### Model calibration

To find model parameters that provide the best description of available experimental data, we used the following model calibration algorithm.

Step 1. Assume all tubulins are in one nucleotide state (GTP hydrolysis turned off). Set a plausible PF bending stiffness from the range 35 to 200 kcal mol^−1^ rad^−2^ (based on Fig. 1f).

Step 2. Use the yeast tubulin association rate constant *k*_*on*_ = 0.26 µM^−1^ s^−1^ per PF as the best starting value for *k*_*on*_^50^. This *k*_*on*_ is adjusted, if necessary, at Step 5.

Step 3. Simulate MT growth at 10 µM tubulin for a set of *a*_*lat*_ values. For each *a*_*lat*_ vary *b*_*lat*_ and *b*_*long*_ to find combinations of {*a*_*lat*_*, b*_*lat*_*, b*_*long*_} that describe:

(A) MT disassembly: MT shortening rate of ~ −400 nm s^−1^; PF curl length in the range between 40 and 80 nm^25,44^;
(B) MT assembly: MT growth rate of ~20 nm s^−1^; PF curl length in the range between 15 and 40 nm^16,25,46,49^.

Step 4. With each of the combinations of {*a*_*lat*_*, b*_*lat*_, *b*_*long*_} for MT disassembly from Step 3(A), simulate MT shortening under opposing load and determine the combination of {*a*_*lat*_, *b*_*lat*_, *b*_*long*_}, which provides a correct stall force of about 30 pN^10,11,28,29^. If successful, use this parameter set as *default for MT shortening* and proceed to the next step. If not, return to Step 1.

Step 5. Keeping the *a*_*lat*_ determined at Step 4, simulate MT growth with a set of tubulin concentrations, varying {*b*_*long*_, *b*_*lat*_} in the vicinity of the values that describe MT assembly (Step 3(B)). Identify those {*b*_*lat*_, *b*_*long*_} which lead to a critical tubulin concentration in the range of 0–2 µM^16,46,49^; adjust the slope of the MT growth rate dependence on tubulin concentration to match experimental data by re-defining *k*_*on*_ and *c*_tub_, keeping their product unchanged. If successful, use this parameter set as *default for MT growth*.

Other model parameters were set based on previous studies and not investigated in detail in this work (Supplementary Tables 1 and 2). Using the above algorithm, we found that the model can be calibrated with PF bending stiffness of 78–174 kcal mol^−1^ rad^−2^. Lower bending stiffness values do not support long-lasting tip tracking of Dam1 ring with the depolymerizing MT end under high forces, as the Dam1 ring slips off the soft PFs.

### Simulations of MT behavior under mechanical load

To simulate MT growth under opposing force, we represented the flat surface of the obstacle by a large sphere, with 5 µm diameter. The obstacle sphere was described analogously to a Dam1 subunit, but it was constrained to move along the MT axis. To take into account possible effects of the obstacle on the tubulin on-rate, we only allowed those tubulin incorporations that corresponded to arrival at positions where tubulin dimers would not overlap with the obstacle.

In simulations of MT growth and shortening under force transduced via a circular coupler, the Dam1 ring complex was modeled as a set of 13 connected spheres with flexible linkers that could interact with tubulin subunits, like in our previous study^9^. The centers of Dam1 subunits were constrained to lie in the planes of the corresponding PFs, so each subunit was described with two positional and one angular coordinate. The center of the Dam1 ring could only move along the MT axis. The potential energy of Dam1−tubulin interaction contained two terms: a repulsive term $\rho \left( d \right)$, as in the case of single PF simulations (Eq. 3), and an attractive term $\varepsilon \left( d \right)$, describing the interaction between a flexible C-terminal linker of Dam1 subunit with tubulin surface. The latter term was introduced as follows:

10 $$\varepsilon \left( d \right) = \left\{ {\begin{array}{*{20}{l}} { - E + \frac{1}{2}k_{{\it{{linker}}}}^{{\it{{Dam1}}}}\left( {l_0 - d} \right)^2,} \hfill & {{\mathrm{{if}}}\,l_0 \,\le \,d \,\le\, l_{{\it{{max}}}}} \hfill \\ { - E,} \hfill & {{\mathrm{{if}}}\,R + r \,< \,d \,< \,l_0} \hfill \\ {0,} \hfill & {{\mathrm{{if}}}\,d \,> \,l_{{\it{{max}}}}} \hfill \end{array}}, \right.$$

where *d* is the current distance between the center of Dam1 subunit and the closest tubulin subunit, *l*_0_ = 5 nm is the equilibrium linker length, *l*_*max*_ = 6 nm is the maximal linker length, $k_{{\it{{linker}}}}^{{\it{{Dam1}}}}$ = 0.01 kcal mol^−1^ nm^−2^ is stiffness of the linker, $E = \frac{1}{2}k_{{\it{{linker}}}}^{{\it{{Dam1}}}}\left( {l_{{\it{{max}}}} - l_0} \right)^2$ is the depth of Dam1−tubulin interaction potential. Linkers of Dam1 subunits formed contacts with the closest tubulin subunits and continued interacting with that given subunit until the energy of attraction *ε*(*d*) turned to zero due to stretching of the linker. After that a new contact with the most proximal subunit could be formed, if any tubulin subunit was sufficiently close for the linker to reach it.

### Growth and imaging of MTs

Pure porcine tubulin from Cytoskeleton (Denver, CO Cat. # t238p) was polymerized onto the MTs of axonemes prepared from *Chamydomonas* flagella^75^, a generous gift from Mary Porter, University of Minnesota. Isolated axonemes were diluted into a buffer containing 80 mM 1,4-Piperazinediethanesulfonic acid (PIPES) at pH 6.9 supplemented with 1 mM GTP, Ethylene-bis(oxyethylenenitrilo)tetraacetic acid (EGTA), and MgCl_2_ (BRB80), then applied as a 2–3 μl drop to a C-flat holy, carbon-coated electron microscope grid (4-µm diameter holes separated by 2 µm) (Electron Microscope Sciences, Hatfield, PA), which had recently been glow-discharged. Axonemes were given 30 s at room temperature to attach to the carbon film, then excess fluid was blotted away. For most experiments, the commercial tubulin at a nominal concentration of 100 µM was diluted to the stated concentrations with BRB80 at 0 °C. α1B/βI+βIVb tubulin was purified from tsA201 cells as described previously^54^. Cells were lysed by gentle sonication in BRB80 pH 6.8 (80 mM PIPES, 1 mM MgCl_2_, 1 mM EGTA), 1 mM dithiothreitol, and 25 µg/ml benzonase. The lysate was cleared by ultracentrifugation at 444,000 × *g* for 15 min at 4 °C. The homogenate was loaded onto a NHS-column (GE Healthcare) coupled to TOG1. The tubulin was eluted with BRB80 pH 6.8 supplemented with 0.5 M ammonium sulfate and was buffer exchanged using a PD-10 column (GE Healthcare) into BRB80 pH 6.8, 10% glycerol, and 20 µM GTP and was flash frozen in liquid nitrogen. The tubulin was further purified by cycling. Tubulin was buffer exchanged using a PD10 column into 1XBRB80 and 20 µM GTP and flash frozen in liquid nitrogen. Mass spectrometric analysis of this tubulin indicated that it contains one major α-tubulin (α1B) and two β-tubulin (βI+βIVb) isoforms (Supplementary Fig. 3c). In experiments with MT-targeting drugs we used tubulin prepared following a published protocol^76^. Briefly, porcine brain tissue was isolated, cleaned, and homogenized using a stainless-steel blender. The homogenized slurry was clarified by ultracentrifugation at 100,000 × *g* for 1 h. Tubulin heterodimers were purified by the well-characterized double-cycling protocol; microtubules were assembled at 37 °C for 1 h and then the pellet was collected after centrifugation. The collected MT pellet was depolymerized on ice for 1 h, after which the clarified supernatant was collected following another centrifugation. This process was performed twice. The double-cycled protein was further purified by liquid chromatography using a phosphocellulose anion-exchange column^76^. This protein was 2–3× more active in polymerization than the commercial protein, so it was used at one-third the concentration.

For some experiments, tubulin was supplemented with a drug or protein that would modify its rate of polymerization. The concentrations of these supplements are given in the description of each experiment. In all cases, samples were supplemented with a low titer of 10 nm colloidal gold to serve as tilt fiducials during tomographic reconstruction. Five microliters of each polymerization mixture at 0 °C was added to the axoneme-containing surface of the grid before it could dry, then the grid was drawn up into the prewarmed and hydrated chamber of a plunge-freezing device (Vitrobot, FEI ThermoFisher, Hillsboro, OR, or EM GP2 plunge freezer, Leica Microsystems, Buffalo Grove, IL). Samples were incubated in this chamber for 1–8 min at 36 °C and 90% relative humidity. After sufficient time for MT growth, which varied markedly with the conditions used, the grid was blotted with No. 1 filter paper and plunged into liquid ethane. Shortening MTs were made by first growing MTs with the above procedure, then inducing depolymerization by introducing conditions that destabilized the polymers. For example, depolymerization could be initiated by a 25-fold isothermal dilution with BRB80 or by adding 0.5 μl of prewarmed MgCl_2_ at 200 mM in BRB80 to the 5 μl drop of polymerization mixture already on the grid.

To obtain a catalyst that would increase the rate of MT elongation, we used the plasmid TOG1-TOG2-myc-6xhis (pPW261), a generous gift from Per Widlund (University of Gothenberg, Sweden) to express a polypeptide from XMAP215 that has been demonstrated to speed tubulin polymerization in vitro in a concentration-dependent manner^51^. The plasmid was expressed in Rosetta (DE2) cells and purified from cell extracts by standard methods. Briefly, cells transfected with the plasmid were grown in LB at 37 °C under Kan/Chlor selection, harvested by centrifugation and lysed by sonication in the presence of lysozyme with 10 mg ml^−1^ pepstatin and PMSF plus a tablet of cOmplete protease inhibitors (Millipore-Sigma, Darmstadt, Germany). TOG-domain protein was initially purified from cytoplasmic extract on a 2 ml nickel column by elution with 300 mM imidazole. The eluent was further purified on a HiTrap SP HP column (GE Healthcare, Uppsala Sweden), using the cation exchange buffers recommended by the manufacturer. Purity and concentration of the final product were assessed by SDS gel electrophoresis and staining with Coomassie blue, calibrated by running standards of known amounts of bovine serum albumin in the same slab gel and measuring the amounts of dye by scanning densitometry. The protein used was ~98% pure.

Grids frozen as described above were transferred to liquid nitrogen and kept under this liquid, either for storage and later use or for immediate use by transferring them to a cryo-transfer holder (Gatan Inc., Pleasanton, CA, model 910) and then inserting into a Tecnai F20 electron microscope from FEI ThermoFisher (Hillsboro, OR) for examination at <−170 °C. Data were collected using a Gatan Ultrascan CCD or Gatan K3 direct detector. Growing MTs were identified as extensions from axoneme doublet A-tubules; polymer ends that lay over holes were imaged with a bidirectional tilt series about a single axis, ranging first from 0° to about +60°, then from 0° to −60°. Microscope magnification was set to let the camera sample the specimen with 0.9 nm pixels. Images in a tilt series were collected at 2° tilt increments with a defocus of −4 µm; low-dose imaging conditions were employed, so the total electron dose for a tilt series was ~80e A^−1^. These images were used for 3D reconstruction by back-projection using the IMOD program suite^77^, followed by filtering with nonlinear, anisotropic diffusion, as implemented in the same package.

### Tracing PFs

The “slicer” window of 3dmod was used to orient and position the axis of each MT collinear with the vertical axis of the viewing window. The slicer window was then tipped 90° about the horizontal axis, so the MT was viewed in cross section. From this orientation, the origin of the coordinate system could be placed at the center of the MT with considerable precision. Now the slicer window was returned to an orientation parallel to the MT axis, and a 2–4-nm-thick sampling lamina was rotated about this axis, displaying the MT walls on either side of the rotation axis at each azimuthal orientation^25^. A contour was drawn on each PF, both where it ran straight along the MT wall and where it curved outward from the MT axis. Any place along a possible PF that showed a sharp change in curvature (e.g., 90° in a few pixels) or an obvious change in either thickness or density of stain was interpreted as some non-tubulin material associated with that PF, rather than a continuation of the PF. PFs were sought at azimuthal angles separated by ~27° (360/13), since MTs grown from axonemes contain 13 PFs. If no curving PF was found at an orientation where a PF should be, this position in the MT circumference was represented by a straight line, drawn in the MT wall. The set of all such traces depicted the structure of each MT end. We could never sample the full MT perimeter because of the limited range of tilt angles available for single-axis electron tomography, which results in a “missing wedge” of data. This feature of our imaging procedure increased the point-spread function in the direction along the beam axis, making resolution anisotropic^78^. Nonetheless, we were able to trace 4–10 PFs on most MTs, yielding a clear characterization of their ends.

Drawing graphic objects whose structures accurately reflected the shape of the flaring PFs at the ends of MTs was sometimes straightforward, sometimes difficult. The low signal-to-noise of the low-dose images meant that tracking was sometimes a challenge, particularly at high angles of tilt, where the increase in point-spread along the *Z*-axis leads to a broadening of each image element in that direction. We have previously assessed the accuracy of our traces in several ways, as described in McIntosh et al.^25^. While there may be errors in our traces, the consistency of our methods should make comparisons between the ends of MTs grown under different conditions quite reliable.

### Analysis of PF traces

Coordinates of traced PFs were processed with a custom Matlab 2017 script^25^ to extract PF curl lengths, average curvature and two additional characteristics: raggedness of MT tips and correlations between adjacent PF lengths and curvatures. Coordinates of simulated PFs were treated in the same manner for consistency.

## Supplementary information

Supplementary Information

Peer Review File

Supplementary Movie 1

Supplementary Movie 3

Supplementary Movie 2

Supplementary Movie 4

Supplementary Movie 5

Supplementary Movie 6

Supplementary Movie 7

Supplementary Code

## Data Availability

Data supporting the findings of this manuscript are available from the corresponding author upon reasonable request. A reporting summary for this Article is available as a Supplementary Information file. The source data underlying Figs. 1c, d, f, 2d−g, 3b−e, 4a, b, d, e, 5d−h, 6c, d, g, h, j, 7c, d and 8c, Supplementary Figs. 2a–d, and 3a, b, and 4, Supplementary Table 3 and correlation analyses of PF lengths and curvatures are provided as a Source Data file. Source data are provided with this paper.

## Source data

Source Data
