# Supplementary material for: Mechanisms of microtubule dynamics and force generation examined with computational modeling and electron cryotomography
Source: Nat Commun. 2020 Jul 28;11:3765. doi: 10.1038/s41467-020-17553-2 (PMC7387542; doi:10.1038/s41467-020-17553-2)
Supplement: Supplementary file 1 — Supplementary Information [file 41467_2020_17553_MOESM1_ESM.pdf]

SUPPORTING INFORMATION

Gudimchuk *et al.*, "Mechanisms of microtubule dynamics and force generation examined with computational modeling and electron cryotomography"

SUPPLEMENTARY FIGURES

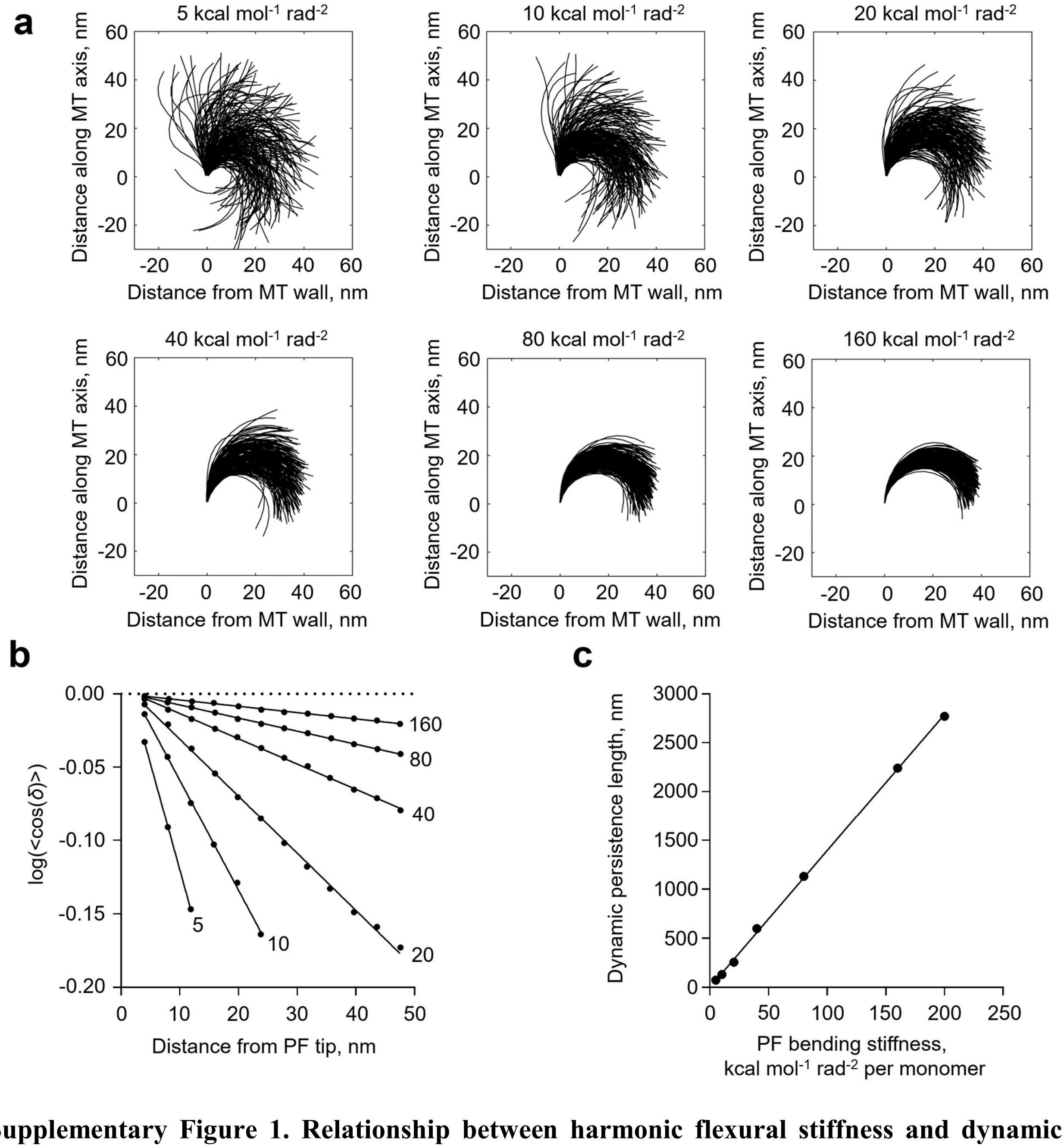

Supplementary Figure 1. Relationship between harmonic flexural stiffness and dynamic persistence length of single PFs

(a) Representative shapes of a hundred configurations of a single PF, simulated with different harmonic bending stiffness: from 5 to 160 kcal mol^{-1} rad^{-2}. (b) Plots of the logarithms of the cosines of mean differences between each measured angle between adjacent line segments and the means of all angles at that distance from the PF tip for each flexural stiffness. Lines are linear fits whose slopes are used for calculating dynamic persistence lengths. Numbers near the curves indicate the harmonic PF bending stiffness coefficient value corresponding to each curve, expressed in kcal

mol^{-1} rad^{-2}. (c) Dependence of dynamic persistence length of simulated PFs on the harmonic stiffness coefficient. Line is a linear fit: Y = 13.96×X.

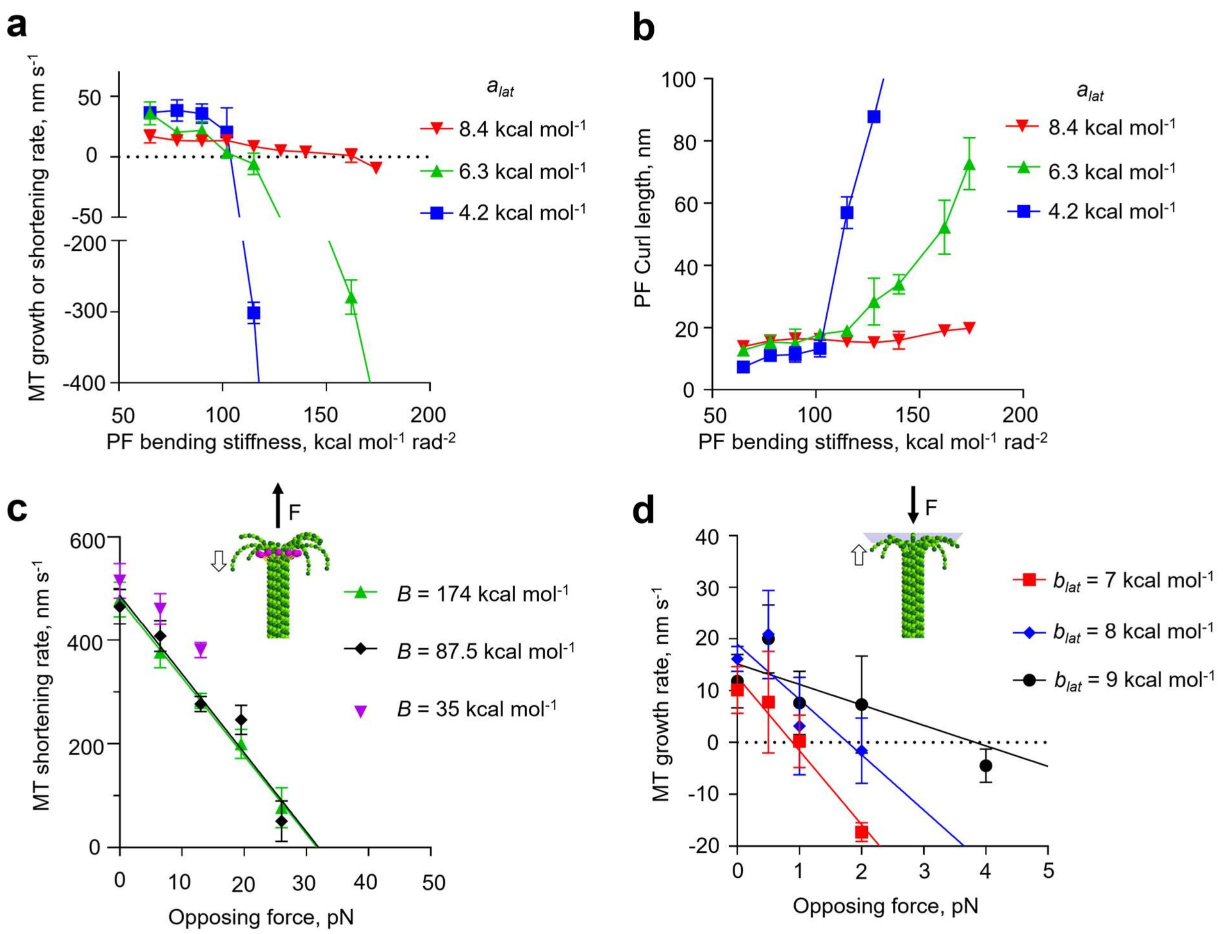

Supplementary Figure 2 Additional analyses of MT behavior in simulations (a) Dependence of MT growth or shortening rate on PF bending stiffness, *B*, for different lateral barrier parameters, a_{lat}. Lateral bond strength, b_{lat}, equals 4.7 kcal mol^{-1}. Numbers less than zero imply shortening. (b) Dependence of PF curl length on PF bending stiffness, *B*, graphed for several lateral activation energies, a_{lat}. (c) Dependence of MT shortening rate on opposing load in simulations with a_{lat} = 6.3 kcal mol^{-1} and three different bending stiffness values. Strengths of lateral bonds (b_{lat}) were calibrated to match an unloaded shortening rate of about 400 nm s^{-1} in each case. At a low PF bending stiffness value (purple dots), the Dam1 ring slipped off the MT end when the applied forces were above 13 pN. (d) Dependence of MT growth rate on opposing loads in simulations with a_{lat} = 6.3 kcal mol^{-1}, 174 kcal mol^{-1} rad^{-2} PF bending stiffness, and three different lateral bond strength values. Lines are linear fits. Data points, describing simulation results in this figure, represent mean ± s.d. based from three repeats of each simulation. Source data are provided as a Source Data file.

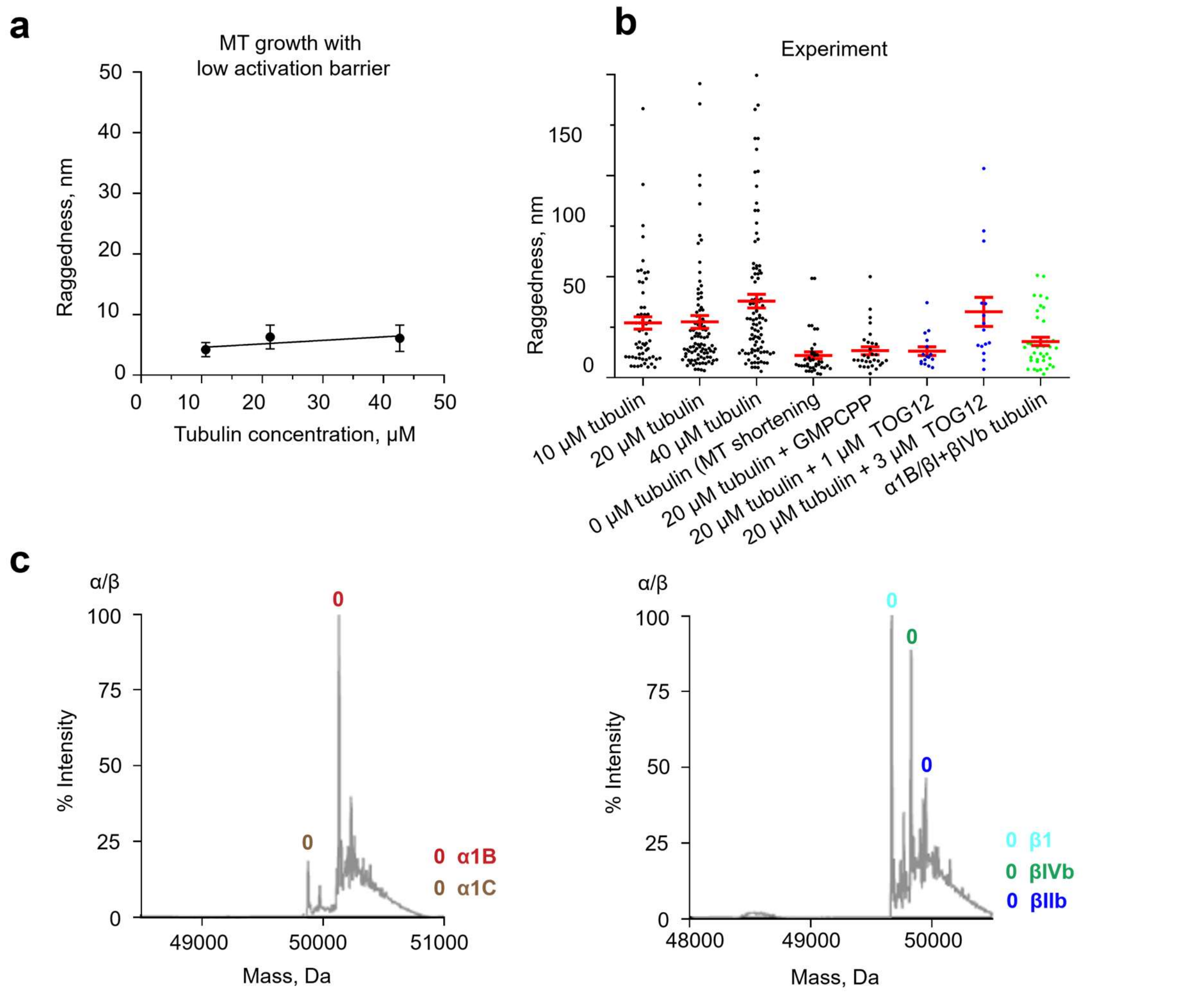

Supplementary Figure 3. Additional data on MT end raggedness. (a) Dependence of MT end raggedness on tubulin concentration in simulations with a low activation barrier a_{lat} = 2.1 kcal mol^{-1}. Each data point shows mean ± s.d. based on three independent simulation runs. Line is a linear fit. (b) Scatter plots of experimentally observed MT end raggedness under different conditions. Source data are provided as a Source Data file. (c) Mass-spectra of α1B/βI+βIVb tubulin.

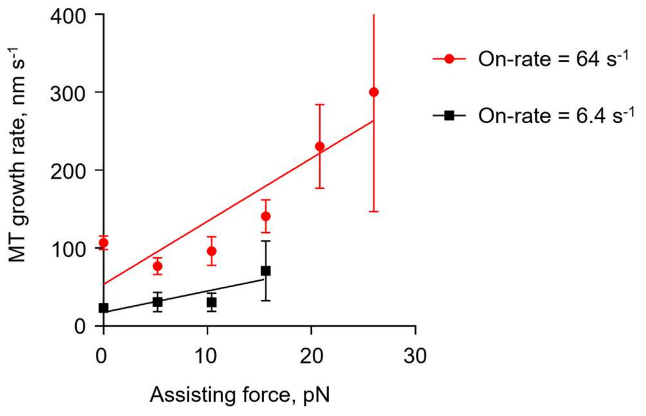

Supplementary Figure 4. MT growth acceleration by assisting force for two tubulin on-rates Each data point shows mean ± s.d. based on three independent simulation runs. Lines are linear fits. Source data are provided as a Source Data file.

SUPPLEMENTARY TABLES

Supplementary Table 1. Summary of MT model parameters. Default values were used in all simulations unless specified otherwise. *Note that we also considered a scenario of GTP hydrolysis, in which the nucleotide state affected PF bending stiffness rather than the strength of the lateral bond (see Fig. 3d,e and Source Data file for more details).

Parameter	Description	Default value	Reference
r_{lat}	width of energy potential well characterizing the length of lateral tubulin–tubulin bond	0.08 nm	Efremov et al., 2007
a_{lat}	energy parameter for lateral repulsion; roughly equals the height of the activation energy barrier for lateral bond	6.3 kcal mol^{-1}	this work
b_{lat}^{GTP}	depth of the potential well for lateral bonds between GTP-tubulins *	8 kcal mol^{-1}	this work
b_{lat}^{GDP}	depth of the potential well for lateral bonds between GDP-tubulins *	4.7 kcal mol^{-1}	this work
r_{long}	width of energy potential well characterizing the length of lateral tubulin–tubulin bond	0.13 nm	Zakharov et al., 2015
a_{long}	energy parameter for longitudinal repulsion; roughly equals the height of the energy barrier for longitudinal interdimer bond	4.5 kcal mol^{-1}	Zakharov et al., 2015
b_{long}	depth of the potential well for longitudinal interdimer bond	10 kcal mol^{-1}	this work
k	stiffness of longitudinal intradimer bond	310 kcal mol^{-1} nm^{-2}	Zakharov et al., 2015
B	PF bending stiffness *	174 kcal mol^{-1} rad^{-2}	Zakharov et al., 2015, Efremov et al., 2007
θ_0	equilibrium angle between tubulin monomers	0.2 rad	McIntosh et al., 2018
c_{tub}	soluble tubulin concentration	5 - 40 µM	this work
$k_{on,PF}$	on-rate constant for tubulin addition per PF	0.64 µM^{-1} s^{-1}	this work
k_{hydr}	GTP hydrolysis rate constant	0.24 s^{-1}	Maurer et al., 2014
T	temperature	310 K	this work
η	viscosity	0.16 Pa s	Zakharov et al., 2015
dt	time step for dynamic algorithm	50 ps	this work
t_{kin}	time step for kinetic algorithm	13 ms	this work

Supplementary Table 2. Summary of additional model parameters, describing pushing and pulling force simulations

Parameter	Description	Value
x	distance between MT wall to the center of Dam1 subunit in single PF simulations	7 nm
k_{rep}	stiffness of tubulin-Dam1 subunits repulsion, restricting their penetration into each other	100 kcal mol^{-1} nm^{-2}
r	radius of tubulin subunit	2 nm
R	radius of Dam1 subunit	3 nm
l_0	equilibrium Dam1 linker length	5 nm
l_{max}	maximal Dam1 linker length	6 nm
k_{linker}^{Dam1}	stiffness of Dam1 linker	0.01 kcal mol^{-1} nm^{-2}
k_{lat}^{Dam1}	stiffness of lateral Dam1 subunits interaction in the ring	100 kcal mol^{-1} nm^{-2}
F	constant force, applied to each Dam1 subunit	varied
D	diameter of the sphere, serving as an obstacle for MT growth	10 µm

Supplementary Table 3. Characteristics of PF curls in experiments in vitro. Tubulin concentration is 20 µM unless specified otherwise. Mean ± SD values are shown

Dataset	PF length, nm	PF curvature, deg per dimer	N of PFs
10 µM tubulin	34 ± 13	21 ± 13	374
20 µM tubulin	35 ± 14	21 ± 14	362
40 µM tubulin	39 ± 20	18 ± 13	369
1 µM TOG12	30 ± 13	23 ± 15	137
3 µM TOG12	25 ± 12	25 ± 16	119
10 nM epothilone	25 ± 14	23 ± 15	470
10 nM paclitaxel	21 ± 9	26 ± 14	212
1 µM paclitaxel	38 ± 41	13 ± 14	376
α1B/βI+βIVb tubulin	21 ± 11	28 ± 19	303

Supplementary Table 4. Description of MT assembly/disassembly rates, MT end structures and force development in published models. Only detailed, stochastic, dimer-scale models of MTs are considered. + or – indicate whether this model accounts for the experimental data mentioned. Question marks indicate that the answer is not clear. Asterisks (*) indicate that a particular model was not directly applied to that data type in the original publications, but we have deduced the answer, based on our own analysis. 'n.a.' means *not applicable* to the description of a certain type of experimental evidence. In the case of kinetic Monte-Carlo models, we call the lateral tubulin-tubulin activation energy barrier 'implicit', rather than 'zero' because the rates of lateral bond formation and breakage in those models are low, which corresponds in practice to fairly high activation energy barriers in more detailed models.

Model type	Curvature of GTP-PFs in the model	Straight					Curved		
	Lateral tubulin-tubulin activation energy barrier in the model	Implicit		Zero	High		Implicit	Low	High
	Example references	Chen and Hill 1985; Bayley et al., 1990; Martin et al., 1993	VanBuren 2002; Piedra et al., 2016	VanBuren 2005; Schek et al., 2007	Efremov et al., 2007	Zakharov et al., 2015	Margolin et al., 2012	This work (low activation barrier)	This work (high activation barrier)
Experimental data	Dependence of MT growth rate on soluble tubulin concentration	+	+	+	n.a	+	+	+	+
	Independence of MT shortening rate from soluble GTP-tubulin concentration	?	+*	?	+*	+	+*	+	+
	Curvature and length of PF curls at growing MT ends	-	-	-	n.a	-	+	+	+
	Curvature and length of PF curls at shortening MT ends	-	+*	+*	+	+	+*	+	+
	Generation of pushing force by growing MT	n.a.	n.a.	+	n.a.	+*	n.a.	+	+
	Generation of pulling force by shortening MT	n.a.	n.a.	-*	+	+*	n.a.	-	+
	Dam1 coupling with growing MT ends under assisting force, and acceleration of MT growth by assisting force	n.a.	n.a.	-*	n.a.	-*	n.a.	-	+

Description of the code's functionality

The *execute()* function describes the main stages of a computational step of the Brownian dynamics model of MT. The pseudocode below shows which computational steps are made in parallel, and at which points the computations are synchronized.

```
function execute():
    for proto in protofilaments:
        tasks_gradient.add(TaskLateralRight(proto))
    for proto in protofilaments:
        tasks_gradient.add(TaskLongitudal(proto))
    for proto in protofilaments:
        tasks_gradient.add(TaskLateralLeft(proto))

    for proto in protofilaments:
        tasks_move.add(TaskMoveDepolymerize(proto))
    for proto in protofilaments:
        tasks_move.add(TaskFillRandom(proto))

    #start of OpenMP parallel section
    counter = start
    while counter < finish:
        for task in gradient_tasks_for_this_worker:
            task.run()
        wait other workers

        counter = counter + 1

        for task in move_tasks_for_this_worker:
            task.run()
        wait other workers

        #start of section executed only by master worker
        if  not (counter % step_polymerization):
                polymerization()
        if  not (counter % step_hydrolysis):
                hydrolysis()
        if  not (counter % check_overcurled_rate):
                overcurled()
        if  not (counter % update_frame_rate):
                update_frame()
        if  not (counter % write_snapshot_rate):
                snapshot(counter)
        #end of section executed only by master worker

        #wait other workers
    #end of parallel section
```

protofilaments is an array of 13 tubulin PFs

tasks_gradient – computes energy gradients
tasks_move – changes tubulin monomer coordinates

TaskLateralRight – fills the lateral component of the energy gradient, corresponding to the interaction with the right tubulin neighbor.

TaskLongitudal – fills the longitudinal component of the energy gradient

TaskLateralLeft – fills the lateral component of the energy gradient, corresponding to the interaction with the left tubulin neighbor.

TaskMoveDepolymerize – changes the PF coordinates, removing depolymerized PF fragments. A tubulin dimer and a PF fragment above it are considered depolymerized if the distance between the dimer's bottom interaction point and the upper interaction point of the underlying dimer exceeds *deploly_cutoff*

TaskFillRandom – fills an array with pre-computed random numbers, which are used for updating PF coordinates

snapshot() – records the current MT configuration to a file

```
function polymerization():
   for proto in protofilaments:
      if get_uniform_random_value() < polymerization_probability:
         proto.add_dimers_back(1, DimerTypeT, teta0_T)
```

proto.add_dimers_back(dimer_count, dimer_type, angle_with_prev) – adds *dimer_count* dimers of *dimer_type* type with *angle_with_prev* angles the to the plus-end of the PF, on top of the terminal dimer.

```
function hydrolysis():
   for proto in protofilaments:
      for c = 0; c < proto.monomers.size(); c += 2:
         mono = proto.monomers[c]
         mono_next = proto.monomers[c + 1]
         if mono.type == DimerTypeT and get_uniform_random_value() <=
hydrolysis_probability:
            mono.type = DimerTypeD
            mono_next.type = DimerTypeD

      for n in proto.hidden_gtp_mono:
         if n % 2 and get_uniform_random_value() <= hydrolysis_probability:
            proto.hidden_gtp_mono.remove(n)
            proto.hidden_gtp_mono.remove(n – 1)
```

proto.hidden_gtp_mono is a list of "hidden" GTP-tubulin monomers, whose nucleotide states and positions within MT lattice are remembered, but the coordinates are not explicitly updated, because they are not within the 'modeling frame'.

The *overcurled()* function serves for removing the tubulin monomers from the PF curl, in case if the curl has made a full 360 degrees turn. This function is needed to speed up code execution when the simulation is performed with the model parameters, leading to a very fast depolymerization and long PF curls.

```
function overcurled():
    threshold = 2 * pi
    for i = 1; i < proto.monomers.size(); ++i:
        mono = proto.monomers[i]
        if mono.y_rot_angle >  threshold:
            if i % 2:
                proto.remove_monomers(i - 1, proto.monomers[i].size())
            else:
                proto.remove_monomers(i, proto.monomers[i].size())
```

The *update_frame()* function speeds up the simulation by excluding the plus-end-distal parts of PFs from explicit simulation (they are considered static). Only the dynamics of the plus-end-proximal tubulin dimers, located within the 'modeling frame' are computed. The modeling frame shifts in the plus-end direction when the MT grows. During MT shortening, the modeling frame shifts in the minus-end direction, while new layers of previously static MT lattice are introduced into the simulation. This allows efficient modeling of MT growth and shortening in a wide range of MT lengths.

```
function update_frame():
    min_inclinated = get_number_to_equilibrium(protofilaments)

    shift_value = 0
    if min_inclinated >= (first_fixed + 2 * min_free_length) or
        min_inclinated < (first_fixed + min_free_length):
        shift_value = (first_fixed + 1.5 * min_free_length) / 2 – min_inclinated / 2
    else:
        return

    if shift_value == 0:
        return

    if shift_value > 0:
        for proto in protofilaments:
            proto.add_dimers_front(shift_value)
    else:
        for proto in protofilaments:
            for i = 0; i < 2 * shift_value; ++i:
                mono =  proto.monomers[i]
                if mono == DimerTypeT:
                    proto.hidden_gtp_mono.insert(mono.mono_number)
            for i = 0; i < 2 * shift_value; ++i:
                proto.monomers.remove(0)
```

By default, *first_fixed* equals 2 tubulin monomers, *min_free_length* equals 4 tubulin monomers. This defines the modeling frame: (*first_fixed* + *min_free_length*, *first_fixed* + 2**min_free_length*) = (6, 10). *get_number_to_equilibrium()* finds the position of the terminal monomer of the shortest straight PF or the shortest start position of PF curl. If this position is outside of the modeling frame, the algorithm moves the modeling frame, to the center of that position.
