# Supplementary material for: Mechanisms of microtubule dynamics and force generation examined with computational modeling and electron cryotomography
Source: Nat Commun. 2020 Jul 28;11:3765. doi: 10.1038/s41467-020-17553-2 (PMC7387542; doi:10.1038/s41467-020-17553-2)
Supplement: Supplementary file 10 — Supplementary Code [file 41467_2020_17553_MOESM10_ESM.zip › BD_MT_model/README.pdf]

Brownian dynamics model of the microtubule

Overview

This code implements Brownian dynamics simulations of tubulin protofilament (PF) mechanics and microtubule (MT) assembly/disassembly process, described in Gudimchuk et al., 2020. The following code modifications are available:

(i) simulation of a single PF;
(ii) simulation of a MT in a force-free regime
(iii) simulation of a MT growing against an obstacle (opposing load),
(iv) simulation of a MT growing or shortening under a force mediated via Dam1 ring

Contents

The software package contains

- three modifications of the source code (subfolders mtrestart_plane, mtrestart_pure, mtrestart_ring)
- a Demo folder, including four sets of files to run examples of different simulation setups
- common.py, animation.py – python scripts for visualization of simulation results
- README file with instructions
- LICENSE file

System Requirements

<u>Hardware</u>

The software will run even any standard desktop PC (e.g. RAM: 2 GB, CPU: 1 core, 2.5 GHz).

For production simulation runs (~ seconds of MT dynamics), using multithreaded processors (thread count > 16) is recommended.

About 1 GB of hard drive storage space is required per 1 second of a typical simulated MT trajectory (if the MT configuration snapshots are recorded once per 10^7 modelling steps).

<u>Software</u>

The code has been developed for Linux operating systems and tested on Ubuntu 16.04

Other required software:

- gcc compiler with C++11 support (e.g. gcc 5.4.0)
- cmake (version 2.8 +)

(Optional): for visualization:

- Python 2.7 with the following modules:

numpy
mayavi
matplotlib

Installation

Build an executable file, using cmake:

for simulation of single PF or whole MT in a force-free regime, use
mtrestart_pure/CMakeLists.txt

for simulation of MT growing against an obstacle (opposing load):
mtrestart_plane/CMakeLists.txt

for simulation of MT growing or shortening under a force mediated via Dam1 ring:
mtrestart_ring/CMakeLists.txt

Building executable files should take 1 - 3 minutes with a standard computer. An executable file 'MTRestart', 'MTRestart_plane' or 'MTRestart_ring' will be created in the '../build/src/' subdirectory of the directory, containing the respective code. Also, an executable file 'SingleProtofilament' (for simulation of single PF) will be created in '../build/single_protofilament/' directory.

Running the simulations

Create a **conf.txt** file and describe model parameters in the following format:

parameter1 value
parameter2 value
…
parameterN value

The following set of configurable parameters is available:

K_hydrolysis – hydrolysis constant, s^{-1}
K_on – tubulin association rate per MT, $\mu M^{-1} \cdot s^{-1}$
T – temperature, K
a_lat – 2 × lateral activation energy barrier, $kcal \cdot mol^{-1}$
a_long - 2 × longitudinal activation energy barrier, $kcal \cdot mol^{-1}$
b_coeff_D – PF bending stiffness coefficient for GDP-tubulins, $kcal \cdot mol^{-1} \cdot rad^{-2}$
b_coeff_T - PF bending stiffness coefficient for GTP-tubulins, $kcal \cdot mol^{-1} \cdot rad^{-2}$
b_lat_D – lateral bond strength for GDP-tubulins, $kcal \cdot mol^{-1}$
b_lat_T – lateral bond strength for GTP-tubulins, $kcal \cdot mol^{-1}$
b_long – longitudinal bond strength, $kcal \cdot mol^{-1}$
concentration – tubulin concentration, μM
dynamic_viscosity – water viscosity, Pa·s
force – force applied to each Dam1 subunit or the obstacle, N
harmonic_interdimer – (technical): Boolean variable that allows switching from interdimer potential with an activation barrier to the non-breakable harmonic potential, 'true' / 'false'
k_coeff – intradimer stiffness coefficient, $kcal \cdot mol^{-1} \cdot nm^{-2}$
r0_lat –– parameter defining the width of longitudinal energy potential, nm
r0_long – parameter defining the width of longitudinal energy potential, nm

repulsion_coeff – (technical): stiffness of protein-protein repulsion to prevent penetration, kcal·mol^{-1}·nm^{-2}
sphere_radius – radius of Dam1 subunit, nm
sphere_x – distance between the center of the straight PF and the Dam1 subunit in single PF simulation, nm
steps – simulation duration, # of steps
teta0_D – the equilibrium angle for GDP-tubulin, rad
teta0_T – the equilibrium angle for GTP-tubulin, rad
update_frame_rate – period between modeling frame updates, # of steps
write_snapshot_rate – period of recording MT configurations to a file, # of steps

If some model parameter(s) is (are) not explicitly specified in the **conf.txt** file, default value(s) will be used in the simulation. All model parameter values will be listed in the **log.txt** file, generated as one of the simulation outputs.

The initial coordinates and nucleotide states (GTP vs GDP) of tubulin monomers can be specified in one of the two ways:

1. To start from a blunt-ended MT, entirely in the GDP-state or entirely in the GTP-state, use the following commands, respectively:

   ./MTRestart -c ./conf.txt -t D
   ./MTRestart -c ./conf.txt -t T

2. To start from an arbitrary configuration, with user-defined coordinates and nucleotide states of all tubulin monomers, prepare a **start_snapshot.txt** file and use the following command to launch the simulation:

   ./MTRestart -c ./conf.txt -s ./start_snapshots.txt

start_snapshot.txt file should contain coordinates of tubulin monomers in the same format as the output file **snapshots.txt,** described below.

As a result of the simulation, output files **snapshots.txt** and **log.txt** will be generated in the **_results** folder. The **snapshots.txt** file contains modeling system's coordinates at each recorded simulation time point in the following format:

snapshot *X* started	% number of the time point, corresponding to the snapshot
proto *X* z_angle *X* iteration *X*	% PF number; z_angle defines PF plane orientation, curr. modeling step number
proto *X* mono *X*	% PF number, monomer number in this PF
y_rot *X* z_rot *X* type *X*	% monomer rotation angles about y and z axes, nucleotide (D or T)
center x *X* y *X* z *X*	% coordinates of center of mass of the monomer
radii *X*	% (technical): distance between MT axis and the center a tubulin monomer
right force point x *X* y *X* z *X*	% coordinates of the right interaction site on tubulin monomer surface
left force point x *X* y *X* z *X*	% coordinates of the left interaction site on tubulin monomer surface
up force point x *X* y *X* z *X*	% coordinates of the top interaction site on tubulin monomer surface
down force point x *X* y *X* z *X*	% coordinates of the bottom interaction site on tubulin monomer surface
force sphere center x *X* y *X* z *X* radius *X*	% (only for Dam1 ring simulations): coordinates of Dam1 ring center and radius
force sphere center theta *X* xi *X*	% (only for Dam1 ring simulations): angle coordinates of Dam1 ring
force sphere left point x *X* y *X* z *X*	% (only for Dam1 ring simulations): coord. of Dam1 subunit left interaction site
force sphere right point x *X* y *X* z	% (only for Dam1 ring simulations): coord. of Dam1 subunit right interaction site
force sphere connected *X*	% (only for Dam1 ring sim., techn.): monomer #, to which Dam1 is attached
...etc (for all PFs)...	
plane height *X*	% (only for simulations with an obstacle), position of the surface of the obstacle
snapshot *X* finished	% number of the time point, corresponding to the snapshot

Log.txt file contains information about the version of the code and simulation parameters.

Typically, simulation of 1 second of MT assembly with Intel(R) Core(TM) i7-5820K CPU @ 3.30GHz computer takes about 3.5 days. The simulation uses all available threads (*omp_get_num_procs()*).

Visualization of simulation results

To visualize, use **animation.py** script, which can be launched with the following command:

python animation.py <path_to_file/snapshots.txt>

Execute python animation.py –h for help:

```
usage: animation.py [-h] [-o [OUTPUT]] [-s [SPARSING]] [--azimuth [AZIMUTH]]
                    [--max_frames [MAX_FRAMES]] path_to_snapshots

plot animation from snapshots in directory

positional arguments:
  path_to_snapshots          path to snapshot dir

optional arguments:
  -h, --help            show this help message and exit
  -o [OUTPUT], --output [OUTPUT]
                        path to output file
  -s [SPARSING], --sparsing [SPARSING] only every s-th snapshot will be included in animation
  --azimuth [AZIMUTH]   camera z angle rotation value
  --max_frames [MAX_FRAMES] max frames in animation
```

Demo

The package includes four examples of short simulations to illustrate different modeling setups

Example 1. Single Protofilament

Build project using mtrestart_pure/CMakeLists.txt, then launch the executable file with the following command:

./SingleProtofilament -c ./conf_demo1.txt

This example will be using demo parameters from the **conf_demo1.txt file**, provided with this package.

Simulated time: 10 µs. Estimated run time on a standard machine: ~5 sec

For visualization of results use **animation.py** and command:

python animation.py --azimuth 90 <path_to_file/snapshots.txt>

Example 2. Free MT assembly

Build project using mtrestart_pure/CMakeLists.txt, then launch the executable file with the following command:

./MTRestart -c ./conf_demo2.txt -s ./start_snapshot_demo2.txt % file

This example will be using demo parameters from the **conf_demo2.txt** file and the starting configuration, defined in the **start_snapshot_demo2.txt** file, provided with this package.

Simulated time: 10 µs. Estimated run time on a standard machine: ~2 min

For visualization of results use **animation.py** and command:

python animation.py <path_to_file/snapshots.txt>

Example 3. MT growth against an obstacle (under opposing load)

Build project, using mtrestart_plane/CMakeLists.txt, then launch the executable file using the following command:

./MTRestart_plane -c ./conf_demo3.txt -s ./start_snapshots_demo3.txt

This example will be using demo parameters from the **conf_demo3.txt** file and the starting configuration, defined in the **start_snapshot_demo3.txt** file, provided with this package.

Simulated time: 10 µs. Estimated run time on a standard machine: ~5-10 min

For visualization of results use **animation.py** and command:

python animation.py <path_to_file/snapshots.txt>

Example 4. Disassembly under opposing load, mediated via Dam1 ring

Build project, using mtrestart_ring/CMakeLists.txt, then launch the executable file using the following command:

./MTRestart_ring -c ./conf_demo4.txt -s ./start_snapshots_demo4.txt

This example will be using demo parameters from the **conf_demo4.txt** file and the starting configuration, defined in the **start_snapshot_demo4.txt** file, provided with this package.

Simulated time: 10 µs. Estimated run time on a standard machine: ~5-10min

For visualization of results use **animation.py** and command:

python animation.py <path_to_file/snapshots.txt>
