# Supplementary material for: Mechanisms of microtubule dynamics and force generation examined with computational modeling and electron cryotomography
Source: Nat Commun. 2020 Jul 28;11:3765. doi: 10.1038/s41467-020-17553-2 (PMC7387542; doi:10.1038/s41467-020-17553-2)
Supplement: Supplementary file 2 — Peer Review File [file 41467_2020_17553_MOESM2_ESM.docx]

Peer Review Information

**Journal:** Nature Communications

**Manuscript title:** Mechanisms of microtubule dynamics and force generation examined with computational modeling and electron cryotomography

**Corresponding author name(s):** Dr Nikita Gudimchuk

**Editorial notes:**

**none**

**Reviewer comments & decisions:**

**Reviewer comments, first version:**

Reviewer #1 (Remarks to the Author):

Report on the paper “Mechanisms of microtubule dynamics and force generation examined with computational modelling and electron cryotomography” by Gudimchuk et al.


Gudimchuk et al. studies the effect of protofilament curling on the growth/shrinkage kinetics and force generation in microtubules, using a combination of Monte Carlo and Brownian dynamics simulations, as well as electron cryotomography. The traditional view of microtubule tip structure suggests that growing microtubules are characterised by blunt or pointed ends, while shrinking ones have flared ends. In this “textbook” picture, the hydrolysis of GTP in tubulin to GDP has a central role in the dynamics of microtubules; here, the assumption is that while GTP-tubulin prefers to be “straight” due to energetic reasons , GDP-tubulin prefers a “bent” conformation, as a result of which protofilaments which consist predominantly of GDP-tubulin will have an intrinsic curvature, and hence “peel off” from the microtubule. This instability leads to “catastrophe” in the filament.

In the present paper, motivated by recent experimental observations which showed that both growing and shrinking microtubules have flared tips, the authors challenge this widely prevalent picture. The authors argue that irrespective of the nucleotide state, polymerised tubulin prefers to adopt a bent conformation with respect to its neighbour(s), as a result of which microtubules will generically develop flared tips. The authors then develop a computational model which includes lateral (inter-protofilament) and longitudinal interaction energies between different tubulin subunits, as well as a bending energy, which depends on the deviation in the relative orientation between neighbouring subunits in a protofilament. The model includes a large number of parameters, summarised in two tables in the document “Supporting Information”. While most of them are fixed using data in the literature, estimates for others (e.g., activation barrier for lateral interaction between tubulin dimers) are obtained by comparing the predictions from simulations with existing or current experimental data. The amount of molecular level details used by the authors in the simulations is indeed impressive, which leads to several new insights into microtubule dynamics.

The motivations behind the study are well-stated. The computational part of the study is also very systematic, rigorous and detailed, although given that the internal dynamics of polymerised tubulin in the microtubule is simulated using Brownian dynamics, it is somewhat surprising that the binding of tubulin dimers to the protofilament tips is implemented using a Monte Carlo algorithm ( without explicitly including tubulin diffusion in solution). This is most certainly only a minor issue; I presume that the motivation might have been to reduce computation time, and in any case, this simplification is unlikely to affect their broad conclusions in any way. The paper is very well-written, organized appropriately and the conclusions are presented in a clear manner. The predictions are likely to be of general interest to people doing theoretical and experimental work with microtubules. There is sufficient novelty in the approach and the results for the paper to deserve consideration for publication in Nature Communications.

However, there are a few questions and issues which I would like the authors to answer/clarify before I can give further recommendations on this paper.

Major issues:

1. I find it a bit surprising that the authors are completely silent about hydrolysis of GTP, even if, based on the assumptions of their model, they may not foresee a big role for it. It is certainly a fact that mathematical and computational models based on variants of the “GTP cap theory” have been fairly successful in explaining the broad features of experimental data on the dependence of catastrophe frequency on tubulin concentration, and to a lesser extent, on opposing force. How do the authors visualise force-free catastrophes in their model ? This question is relevant because in page 8, paragraph 3, the authors do suggest the possibility of catastrophes under force in their model.

2. The description of the computational model in section “Methods” is confusing. For example, in line 4 of the first paragraph, when the authors say “The spheres were connected…”, I guess they mean the two spheres/tubulin monomers which form a single dimer, and not two spheres which might belong to two different dimers. Likewise, the term “rotation angle” is used to mean two different things here, (a) the angle of rotation of a single sphere/subunit with respect to an axis joining the two longitudinal sites for intra-dimer interaction, and (b) the relative angle of rotation of the axes between two neighbouring dimers in a protofilament ( see Fig 1, b and c). I suggest using the term “bending angle” uniformly for the latter, as is done in Fig 1.

3. Related to the above issue, how is the bending angle between two neighbouring dimers related to the individual angles of rotation of each sphere in these dimers ? This is not clearly stated anywhere in the text, but in the Langevin equation Eq.9, the differentiation is with respect to the latter, while the last term in Eq.8 involves the former. Perhaps an additional schematic diagram which shows all the degrees of freedom in two neighbouring dimers explicitly will be helpful to the reader.


Additional, minor issues to be looked into before the re-submission:

1. Why is the “equilibrium” bending angle fixed at 0.2 degrees ? The authors could think of adding a reference here, or explain the motivations for the same in page 5.
2. Page 7, para 2, last sentence appears incomplete.
3. Page 14, middle paragraph, the sentence starting with “Forces developed by depolymerising MTs were….”, I could not understand what the authors mean here, since the present study too is, by and large, silent about dynamic instability ! (point (i) above). Would the authors like to modify this sentence ?
4. Page 27, line 3, “concertation” should be “concentration”.
5. Fig 3 d in p.41, y-axis label is missing.
6. In the caption of Fig. S1, the second (b) should be (c).
7. In Table S1, does the on rate for tubulin have wrong units ?


Reviewer #2 (Remarks to the Author):

Mechanisms of microtubule dynamics and force generation examined with computational modeling and electron cryotomography

By Gudimchuk et al.

In this paper, the authors have combined reasoning from an insight into the microtubule literature, computational modeling and electron cryotomography to provide a much-improved mechanism on microtubule force generation and dynamics. They present the lateral activation energy barrier in tubulin interactions as a new and important parameter to understand how microtubules can exert force. The modelling makes a compelling argument that fits well with experimental results som of which has remained unexplainable with the previously suggested models of microtubule polymerization. In particular, how the result could be used to explain the mechanisms of TOG polymerases and EB1 plus end binding proteins shows the strength of the proposed model.

The discrepancy between the in vivo and in vitro electron microscopy on microtubule tip structures has been confusing the field for decades. This research clarifies this discrepancy but could do more to highlight and discuss it. This paper will be of wide interest for cell biologists, especially those trying to understand the cytoskeleton and cell division.

I only had one major concern – that all protofilaments examined had more or less the same curved structure. One could have imagined this being caused by an inability to trace individual protofilaments of another morphology with this method, which is at the boundary for what can be seen in unaveraged cryo-ET data. However, the experiment using higher concentration of taxol showed another, longer and less curved protofilament structure, and with that all concerns are satisfied.

Minor points:

1. Add the reference https://rupress.org/jcb/article-lookup/doi/10.1083/jcb.201009037, as they also showed that growing MT ends are flared in vivo. (first line, page 4).
2. P 6. The sentence “Hence, we obtain..” is unclear to me. Please help the reader also to understand why you choose another value (174 kcal/mol/rad2) as a default for the rest of the paper.
3. P 8. Please cite previous in vivo ET papers that show that microtubule tips in physical contact with a membrane have flared ends.
4. P 10. Last paragraph. How does the high taxol concentration relate to previous in vitro studies like the ones where they saw sheet structures? Could this explain the difference in observed microtubule tip structures?
5. P. 11, first paragraph. Please clarify what a “small lateral activation energy” would be possibly with an example from experiments.
6. P. 11 “We tested these predictions..”. This sentence would benefit with a short explanation of how the growing and shrinking dynamic state was ensured experimentally.
7. P. 12 It is unclear why the section with subheading “There is little or no correltation..” is there. An introduction to why in the beginning of the section would help.
8. P. 16 first sentence is incomplete ..with a full 3D MT model of ______ should be carried out.
9. P. 31 state which plunge freezer was used.
10. P. 32 typo “at 0 degree C at was”
11. P. 33 Mention which software was used for calculating ET reconstructions and subsequent filtering.
12. Figure 5: In figure 4 the average pf length and standard deviation was displayed in their curvature graphs. This is missing in figure 5 and would be helpful additions.
13. Figure 6: b) indicate time aspect somehow, maybe by adding arrows between the progressing states? E) add a title containing experimental condition above the image, like in figures 4 and 5.


Reviewer #3 (Remarks to the Author):

Review of manuscript “Mechanisms of microtubule dynamics and force generation examined with computational modeling and electron cryotomography” by Gudimchuk and colleagues.

Summary: In this manuscript, Gudimchuk and colleagues use a combination of experiments and modeling to investigate the structure of the MT tip and how it changes in response to applied force and alterations to ambient tubulin concentration. This work builds upon previous work from this team showing that growing microtubules in a range of organisms are usually flared and “ragged,” meaning that typically they have at their tips laterally unbound protofilaments that curve out. This earlier paper was viewed by many (most?) in the community as quite surprising (such tip structures were generally assumed to exist only on depolymerizing ends), so a follow up paper addressing this topic is significant.

A major conclusion of the paper is that the ability of depolymerizing microtubules to generate force (essential for chromosome segregation) depends on the height of the activation energy barrier for interactions between protofilaments. In the Discussion, the authors suggest that a source for this barrier could be charge repulsion from the negatively charged nature of the tubulin subunits. The authors also predict that microtubules will exhibit a load-dependent acceleration in the rate of growth, “providing a role for the flared morphology of growing microtubule ends.” The main support for these intriguing ideas comes from simulations. However, the authors also provide a series of experiments in which they look at how microtubule tip structure changes with tubulin concentration and the presence of agents (regulatory proteins, drugs) that alter microtubule dynamics. They use concordance between tip structures in vitro and in silico to support the conclusions of the simulations, and as part of this work, they show that tip structure is “remarkably consistent under various assembly conditions.” These experimental data on tip structures in these different environments will surprise many in the field and are by themselves an important contribution. The conceptual model presented in Figure 7D, which uses these the sum of these ideas to explain the oscillation of metaphase chromosomes is elegant and compelling.

Overall, I think that the topic is appropriate for Nature Communications: the subject is of broad interest, and the results are significant. The work is also technically strong, and the conclusions are well-justified, with one important exception below. Thus, I think that the manuscript has the potential to eventually be published in Nature Communications. However, there are some issues that should first be addressed. Many can be addressed at the level of writing, though it is possible that once clarification is provided, more simulations will be needed. Because this additional information could weaken some of the conclusions, a final determination as to the appropriateness of the work for Nature Communications is not yet possible.


Major concerns:

1. My most significant concern is that the simulations do not appear to include GTP hydrolysis. This is very puzzling to me because the lack of hydrolysis as a process in the simulation seems to undermine the conclusion that the height of the activation energy barrier is important for force generation. Indeed, one might conclude from the present version of this paper that the activation energy barrier is somehow the main source of the energy driving depolymerization. Do the authors mean to imply this? Clearly, force generation requires a source of energy. It seems reasonable to propose that the suggested activation energy barrier contributes to this process. However, given that GMPCPP microtubule do not (to my knowledge) generate force as they depolymerize (or do they?), it seems unreasonable to leave readers with the impression that the activation energy barrier is somehow generating the force. Given all this, how exactly does GTP hydrolysis fit into the conceptual model proposed by the authors?

I can imagine some answers to these questions, so I don’t really think that the (apparent) lack of GTP hydrolysis in the model represents a fatal flaw. However, the presence/absence of GTP hydrolysis in the simulations and the implications of this presence/absence need to be addressed in clear and direct ways. The authors should then integrate this information into their overall conceptual model.


Other concerns:
1. I’m puzzled by the shape of the interaction energy curves. Shouldn’t there be a repulsion as the molecules get too close? There are varied ways to approximate intermolecular interactions, so I’m not worried about this, but please do explain.
2. It would be helpful to the more biological readers if the authors could provide earlier in the manuscript a (brief) discussion of how of how the proposed activation energy barrier might be physically manifested (e.g., through the charge-charge repulsion of the negatively charged PFs as mentioned in the discussion). Similarly, it would helpful if the authors could discuss expected implications of the height of the barrier on the assembly process (e.g., through effects on the rate of lateral bond association and dissociation).
3. I’m puzzled by the data in Figure 1C, which presents the frequency of PF bonds straightening as a function of stiffness, as well as the associated discussion on Page 5. The conclusion here is that “the predicted frequency of PF fluctuations is significantly faster than the rate of arrival and loss of tubulin subunits from the PF tip.” Shouldn’t the rate of bond straightening depend on the length of the PF, especially since the polymerization process is occurring at low Reynolds number? The more I think about this, the more I’m worried that this is a case where the simulation data need to be interpreted very carefully (and any assumptions stated very explicitly) because the physical system could be behaving very differently from the simulated system. Overall, I’m unconvinced of the conclusion above except for the shortest PFs.
4. Concern #3 then raises concerns about the conclusion on page 9 that “MT growth is limited by the rate of new tubulin attachments to the ends of protofilaments, not by the rate of lateral bond closure.” This is an interesting idea, but unless #3 is addressed in a robust way, I think that it needs to be stated much more tentatively.
5. A related issue is that I think that the authors need to incorporate into their discussions the rate of tubulin detachment in their simulations. More specifically, do the authors agree with previous papers that conclude that most incoming tubulin subunits leave before being incorporated into the MT? I assume that they do not. Some explicit discussion of (and perhaps data on) detachment rates would be useful.
6. I’m unconvinced by the argument on Page 7 that when lateral activation energies are low, the model is “overly sensitive” to changes in lateral bond strength. Yes, the authors do use their simulations to show that when the activation energy is low, the system is very sensitive to changes in lateral bond strength. But who is to say that such sensitivity is incorrect? Biology is sometimes poised on a knife edge. Given the large number of regulatory proteins that affect tubulin lateral bond strength, one might even predict a sharp dependence on lateral bond strength.
7. It would be helpful if the authors could compare their “raggedness” measurements to the “taper” measured by other authors.


Other writing issues (mostly minor):
1. The legends are quite spare. It would be helpful if a bit more information could be provided. In particular, it would be helpful if each figure or figure panel could say the data come from. More details about tubulin concentration and other parameters would also be useful.
2. The methods and supplementary data have quite a few typos and/or problems with commas. These sections need some editing to get them up to publication standards.
3. On page 25, please check the value for temperature in following sentence: “Simulations of the tubulin and Dam1 motions were carried out, using equation (9) at T = 0 K.”


Figures:
Figure 1: Nature Communications is a general science journal. Figure 1 should include a cartoon showing how the protofilament visualized fits into the larger structure of a microtubule.

Figure 3: panel D appears to be missing the Y axis label.

**Author rebuttal, first version:**

Reviewer #1 (Remarks to the Author):

Gudimchuk et al. studies the effect of protofilament curling on the growth/shrinkage kinetics and force generation in microtubules, using a combination of Monte Carlo and Brownian dynamics simulations, as well as electron cryotomography. The traditional view of microtubule tip structure suggests that growing microtubules are characterised by blunt or pointed ends, while shrinking ones have flared ends. In this “textbook” picture, the hydrolysis of GTP in tubulin to GDP has a central role in the dynamics of microtubules; here, the assumption is that while GTP-tubulin prefers to be “straight” due to energetic reasons , GDP-tubulin prefers a “bent” conformation, as a result of which protofilaments which consist predominantly of GDP-tubulin will have an intrinsic curvature, and hence “peel off” from the microtubule. This instability leads to “catastrophe” in the filament.

In the present paper, motivated by recent experimental observations which showed that both growing and shrinking microtubules have flared tips, the authors challenge this widely prevalent picture. The authors argue that irrespective of the nucleotide state, polymerised tubulin prefers to adopt a bent conformation with respect to its neighbour(s), as a result of which microtubules will generically develop flared tips. The authors then develop a computational model which includes lateral (inter-protofilament) and longitudinal interaction energies between different tubulin subunits, as well as a bending energy, which depends on the deviation in the relative orientation between neighbouring subunits in a protofilament. The model includes a large number of parameters, summarised in two tables in the document “Supporting Information”. While most of them are fixed using data in the literature, estimates for others (e.g., activation barrier for lateral

interaction between tubulin dimers) are obtained by comparing the predictions from simulations with existing or current experimental data. The amount of molecular level details used by the authors in the simulations is indeed impressive, which leads to several new insights into microtubule dynamics.

The motivations behind the study are well-stated. The computational part of the study is also very systematic, rigorous and detailed, although given that the internal dynamics of polymerised tubulin in the microtubule is simulated using Brownian dynamics, it is somewhat surprising that the binding of tubulin dimers to the protofilament tips is implemented using a Monte Carlo algorithm ( without explicitly including tubulin diffusion in solution). This is most certainly only a minor issue; I presume that the motivation might have been to reduce computation time, and in any case, this simplification is unlikely to affect their broad conclusions in any way.

The Reviewer is correct. We have not explicitly considered the association of tubulin dimers to reduce computation time. We do think that this simplification is unlikely to affect the conclusions of this study in any significant way. Explicit implementation of tubulin dimer association with the microtubule from solution will be an important direction for further work to model the process of spontaneous microtubule nucleation and explicit interaction of tubulins with microtubule associated proteins.

The paper is very well-written, organized appropriately and the conclusions are presented in a clear manner. The predictions are likely to be of general interest to people doing theoretical and experimental work with microtubules. There is sufficient novelty in the approach and the results for the paper to deserve consideration for publication in Nature Communications.

However, there are a few questions and issues which I would like the authors to answer/clarify before I can give further recommendations on this paper.

Major issues:

1. I find it a bit surprising that the authors are completely silent about hydrolysis of GTP, even if, based on the assumptions of their model, they may not foresee a big role for it. It is certainly a fact that mathematical and computational models based on variants of the “GTP cap theory” have been fairly successful in explaining the broad features of experimental data on the dependence of catastrophe frequency on tubulin concentration, and to a lesser extent, on opposing force. How do the authors visualise force-free catastrophes in their model ? This question is relevant because in page 8, paragraph 3, the authors do suggest the possibility of catastrophes under force in their model.

We agree that GTP hydrolysis is a key process, responsible for spontaneous switching between MT assembly and disassembly. The computational expenses associated with simulation of long MT growth times needed to observe catastrophes with a realistic GTP hydrolysis rate constant, precluded us from directly examining catastrophes in the initial version of the manuscript. Although we did not explicitly implement the GTP hydrolysis in the original version of the manuscript, we sought model parametrizations with which MTs would polymerize and other parametrizations with which they would depolymerize. The assumption was that the former

parametrization should correspond to MTs entirely in the GTP-state, while the latter parametrization should describe MTs entirely in the GDP-state. We interpreted the energy difference between the lateral bonds with these two parametrizations as the expected effect of GTP hydrolysis.

To clarify and strengthen this point in the revised version of the manuscript, we have now added new simulations, directly implementing GTP hydrolysis with a realistic rate constant (please, see new Fig 3). As the simulation of only 1 s of MT lifetime requires several days of clocktime in our hardware configuration setup, simulations of hundreds of seconds of MT growth to observe a natural catastrophe could not be performed. Instead, we have simulated a tubulin dilution experiment, which also highlights a distinct, two-state behavior of the MT. To stay open-minded about the possible mechanism of GTP hydrolysis’ effects on tubulins, we considered two possible scenarios: 1) a weakening of the lateral bonds upon GTP hydrolysis; 2) increased rigidity of the PFs upon GTP hydrolysis. With both effects of GTP hydrolysis, we observed a catastrophe a few seconds after the soluble tubulin concentration was set to zero (new videos S3, S4). The resulting simulations show very nicely the way a MT tip changes from net growth to net shortening, while the shapes of the PF at the tip remain largely unchanged. We hope these simulations clarify the role(s) of GTP hydrolysis as the main driver of dynamic instability in our model. Further experiments are required to discriminate between the possible effects of GTP hydrolysis on tubulin’s properties.

2. The description of the computational model in section “Methods” is confusing. For example, in line 4 of the first paragraph, when the authors say “The spheres were connected...”, I guess they mean the two spheres/tubulin monomers which form a single dimer, and not two spheres which might belong to two different dimers. Likewise, the term “rotation angle” is used to mean two different things here, (a) the angle of rotation of a single sphere/subunit with respect to an axis joining the two longitudinal sites for intra-dimer interaction, and (b) the relative angle of rotation of the axes between two neighbouring dimers in a protofilament ( see Fig 1, b and c). I suggest using the term “bending angle” uniformly for the latter, as is done in Fig 1.

We have rewritten the Methods section, implementing these suggestions. Thank you.

3. Related to the above issue, how is the bending angle between two neighbouring dimers related to the individual angles of rotation of each sphere in these dimers ? This is not clearly stated anywhere in the text, but in the Langevin equation Eq.9, the differentiation is with respect to the latter, while the last term in Eq.8 involves the former. Perhaps an additional schematic diagram which shows all the degrees of freedom in two neighbouring dimers explicitly will be helpful to the reader.

We have clarified this issue in the text.

Additional, minor issues to be looked into before the re-submission:

1. Why is the “equilibrium” bending angle fixed at 0.2 degrees ? The authors could think of adding a reference here, or explain the motivations for the same in page 5.

We presume that Reviewer meant radians, not degrees. We have added the references to support that statement.

2. Page 7, para 2, last sentence appears incomplete.

We have changed the grammar of this sentence to make its structure more clear

3. Page 14, middle paragraph, the sentence starting with “Forces developed by depolymerising MTs were....”, I could not understand what the authors mean here, since the present study too is, by and large, silent about dynamic instability ! (point (i) above). Would the authors like to modify this sentence ?

We hope that the new data and discussion of dynamic instability and the role of GTP hydrolysis, which we added in the revised manuscript (see point 1 above), addresses this issue.

4. Page 27, line 3, “concertation” should be “concentration”.
   Fixed. Thank you.
5. Fig 3 d in p.41, y-axis label is missing.
   Fixed. Thank you.
6. In the caption of Fig. S1, the second (b) should be (c).
   Fixed. Thank you.
0. In Table S1, does the on rate for tubulin have wrong units ? Fixed. Thank you.

Reviewer #2 (Remarks to the Author):

Mechanisms of microtubule dynamics and force generation examined with computational modeling and electron cryotomography

By Gudimchuk et al.

In this paper, the authors have combined reasoning from an insight into the microtubule literature, computational modeling and electron cryotomography to provide a much-improved mechanism on microtubule force generation and dynamics. They present the lateral activation energy barrier in tubulin interactions as a new and important parameter to understand how microtubules can exert force. The modelling makes a compelling argument that fits well with experimental results som of which has remained unexplainable with the previously suggested models of microtubule polymerization. In particular, how the result could be used to explain the mechanisms of TOG polymerases and EB1 plus end binding proteins shows the strength of the proposed model.

The discrepancy between the in vivo and in vitro electron microscopy on microtubule tip structures has been confusing the field for decades. This research clarifies this discrepancy but could do more to highlight and discuss it. This paper will be of wide interest for cell biologists, especially those trying to understand the cytoskeleton and cell division.

We are pleased that this reviewer sees our paper as a contribution to the field’s understanding of MT behavior. Our previous publication in the J. Cell Biology was devoted to the differences between our observations and those published by others, so we did not want to belabor those points here. The current version presents the various descriptions briefly, so the context of our current work is clear, but we do not dwell on the issue in a paper that presents a clear and consistent way of looking at the problem.

I only had one major concern – that all protofilaments examined had more or less the same curved structure. One could have imagined this being caused by an inability to trace individual protofilaments of another morphology with this method, which is at the boundary for what can be seen in unaveraged cryo-ET data. However, the experiment using higher concentration of taxol showed another, longer and less curved protofilament structure, and with that all concerns are satisfied.

Minor points:

1. Add the reference [https://rupress.org/jcb/article-lookup/doi/10.1083/jcb.201009037](https://as), as they

also showed that growing MT ends are flared in vivo. (first line, page 4).

Thank you! We have added that reference.

2. P 6. The sentence “Hence, we obtain..” is unclear to me. Please help the reader also to understand

why you choose another value (174 kcal/mol/rad2) as a default for the rest of the paper.

We have rewritten the paragraph on page 6 to explain why we selected that value.

3. P 8. Please cite previous in vivo ET papers that show that microtubule tips in physical contact with a membrane have flared ends.

Thanks. We have now cited Zovko et al. 2008 paper and Rodriguez-Garcia et al., 2020 - a recent publication from M.Dogterom and A. Akmanova laboratories, showing that forces, developed by growing and shortening MTs contribute to membrane remodeling.

4. P 10. Last paragraph. How does the high taxol concentration relate to previous in vitro studies like the ones where they saw sheet structures? Could this explain the difference in observed microtubule tip structures?

The Reviewer raises an important but quite complex point. As s/he notes, the fact that we can see long, gently curving protofilament bundles with our methods is important, since these structures have been reported by others as a common tip feature of growing MTs. Previous reports on the structure of tubulin polymers formed in various taxanes show significant variation in structure (e.g., Jordan et al., Cell, chem boil, 9:93(2002), Fig 3). In our experience, what you see depends in multiple ways on how you look. Samples prepared on conventional, carbon-coated EM grids and imaged after negative staining do show sheets of aligned protofilaments, but such structures are not seen over holes in the sample grid when imaging by cryoEM. We presume, but do not know for sure, that the sheet structures are an artefact of sample flattening when curved tubulin polymers are dried down onto a carbon support film. The relationship between these various Taxol-tubulin polymers and normal microtubules is an issue that will take considerable work to sort out, work that we have not done. We have therefore limited our comments to the

short descriptions currently in the paper. How our observations related to images from the Chretien lab that show predominantly long, gently curving protofilament bundles is currently a matter of speculation, as described with some detail in our recent paper in the J. Cell Biol. We think it is best to minimize its treatment until we know more about the relevant issues.

5. P. 11, first paragraph. Please clarify what a “small lateral activation energy” would be possibly

with an example from experiments.

We have clarified in brackets, what we mean by the small energy barrier.

6. P. 11 “We tested these predictions..”. This sentence would benefit with a short explanation of

how the growing and shrinking dynamic state was ensured experimentally.

We have added this explanation, as suggested by Reviewer

7. P. 12 It is unclear why the section with subheading “There is little or no correltation..” is there.

An introduction to why in the beginning of the section would help.

We have added an introductory sentence, as suggested

8. P. 16 first sentence is incomplete ..with a full 3D MT model of	should be carried out. Fixed. Thank you.
9. P. 31 state which plunge freezer was used.

The freezers used are mentioned explicitly on the next page.

10. P. 32 typo “at 0 degree C at was”
    Fixed. Thank you.
11. P. 33 Mention which software was used for calculating ET reconstructions and subsequent

filtering.

We have added the missing reference.

12. Figure 5: In figure 4 the average pf length and standard deviation was displayed in their

curvature graphs. This is missing in figure 5 and would be helpful additions.

We have now displayed PF lengths with standard deviations in Fig. 5.

13. Figure 6: b) indicate time aspect somehow, maybe by adding arrows between the progressing

states?

Arrows between progressing states were added in Figs. 7 and 8.

e) add a title containing experimental condition above the image, like in figures 4 and 5. Done.

Reviewer #3 (Remarks to the Author):

Review of manuscript “Mechanisms of microtubule dynamics and force generation examined

with computational modeling and electron cryotomography” by Gudimchuk and colleagues.

Summary: In this manuscript, Gudimchuk and colleagues use a combination of experiments and modeling to investigate the structure of the MT tip and how it changes in response to applied force and alterations to ambient tubulin concentration. This work builds upon previous work from this team showing that growing microtubules in a range of organisms are usually flared and “ragged,” meaning that typically they have at their tips laterally unbound protofilaments that curve out. This earlier paper was viewed by many (most?) in the community as quite surprising (such tip structures were generally assumed to exist only on depolymerizing ends), so a follow up paper addressing this topic is significant.

A major conclusion of the paper is that the ability of depolymerizing microtubules to generate force (essential for chromosome segregation) depends on the height of the activation energy barrier for interactions between protofilaments. In the Discussion, the authors suggest that a source for this barrier could be charge repulsion from the negatively charged nature of the tubulin subunits. The authors also predict that microtubules will exhibit a load-dependent acceleration in the rate of growth, “providing a role for the flared morphology of growing microtubule ends.” The main support for these intriguing ideas comes from simulations. However, the authors also provide a series of experiments in which they look at how microtubule tip structure changes with tubulin concentration and the presence of agents (regulatory proteins, drugs) that alter microtubule dynamics. They use concordance between tip structures in vitro and in silico to support the conclusions of the simulations, and as part of this work, they show that tip structure is “remarkably consistent under various assembly conditions.” These experimental data on tip structures in these different environments will surprise many in the field and are by themselves an important contribution. The conceptual model presented in Figure 7D, which uses these the sum of these ideas to explain the oscillation of metaphase chromosomes is elegant and compelling.

Overall, I think that the topic is appropriate for Nature Communications: the subject is of broad interest, and the results are significant. The work is also technically strong, and the conclusions are well-justified, with one important exception below. Thus, I think that the manuscript has the potential to eventually be published in Nature Communications. However, there are some issues that should first be addressed. Many can be addressed at the level of writing, though it is possible that once clarification is provided, more simulations will be needed. Because this additional information could weaken some of the conclusions, a final determination as to the appropriateness of the work for Nature Communications is not yet possible.

Major concerns:

1. My most significant concern is that the simulations do not appear to include GTP hydrolysis. This is very puzzling to me because the lack of hydrolysis as a process in the simulation seems to undermine the conclusion that the height of the activation energy barrier is important for force generation. Indeed, one might conclude from the present version of this paper that the activation energy barrier is somehow the main source of the energy driving depolymerization. Do the authors mean to imply this? Clearly, force generation requires a source of energy. It seems

reasonable to propose that the suggested activation energy barrier contributes to this process. However, given that GMPCPP microtubule do not (to my knowledge) generate force as they depolymerize (or do they?), it seems unreasonable to leave readers with the impression that the activation energy barrier is somehow generating the force. Given all this, how exactly does GTP hydrolysis fit into the conceptual model proposed by the authors? I can imagine some answers to these questions, so I don’t really think that the (apparent) lack of GTP hydrolysis in the model represents a fatal flaw. However, the presence/absence of GTP hydrolysis in the simulations and the implications of this presence/absence need to be addressed in clear and direct ways. The authors should then integrate this information into their overall conceptual model.

We certainly did not mean that the activation energy barrier is the main source of energy for force development or dynamic instability. Rather, we believe that the energy for both processes comes from GTP hydrolysis. The lateral activation energy is needed to ensure that the protofilaments can efficiently use the energy of GTP hydrolysis, accumulated in the form of mechanical strain. As we explained in our response Reviewer 1, who expressed a similar concern, the process of GTP hydrolysis was not considered explicitly in the initial version of the manuscript only because of the computational expenses associated with long simulations needed to observe catastrophes. We did imply that the nucleotide state of tubulins should dictate the behavior of MT polymers. In order to strengthen and illustrate this point, we have now performed simulations of tubulin dilution experiments with explicit random GTP hydrolysis (new Fig 3, videos S3, S4). In those simulations, we considered two possible parametrizations for GTP- and GDP-tubulins, in which the nucleotide defined either the lateral bond strength or PF bending stiffness. Importantly, the lateral activation energy barrier was the same in all of those simulations and it was not modified by GTP hydrolysis in any of the considered scenarios. Both types of simulations recapitulated a delayed catastrophe in tubulin dilution experiments, demonstrating that the GTP cap can protect the MTs from disassembly until it is lost due to hydrolysis of tubulin dissociation. Likewise, we suggest that GTP hydrolysis should serve as a major driver of MT dynamic instability in presence of soluble tubulin.

Other concerns:

1. I’m puzzled by the shape of the interaction energy curves. Shouldn’t there be a repulsion as the molecules get too close? There are varied ways to approximate intermolecular interactions, so I’m not worried about this, but please do explain.

Our graphs may seem a bit confusing or unusual because, in contrast to common practice, we represent the interaction energy curves as a function of the distance between interaction points, located on the surface of tubulins, rather than as a function of the distance between their centers of mass. The repulsive part of the curve in the latter representation implies that molecules start penetrating into each other when the distance between their centers of mass come closer to each other than the sum of their radii. In our case, the potential is spherically symmetric, so if the tubulins overlap, they do repel each other because the lowest energy state corresponds to the configuration when the distance between the interaction points on their surfaces is zero.

2. It would be helpful to the more biological readers if the authors could provide earlier in the manuscript a (brief) discussion of how of how the proposed activation energy barrier might be

physically manifested (e.g., through the charge-charge repulsion of the negatively charged PFs as mentioned in the discussion). Similarly, it would helpful if the authors could discuss expected implications of the height of the barrier on the assembly process (e.g., through effects on the rate of lateral bond association and dissociation).

We have added a brief discussion of the possible nature and effects of the activation energy barrier, as suggested by the Reviewer.

3. I’m puzzled by the data in Figure 1C, which presents the frequency of PF bonds straightening as a function of stiffness, as well as the associated discussion on Page 5. The conclusion here is that “the predicted frequency of PF fluctuations is significantly faster than the rate of arrival and loss of tubulin subunits from the PF tip.” Shouldn’t the rate of bond straightening depend on the length of the PF, especially since the polymerization process is occurring at low Reynolds number? The more I think about this, the more I’m worried that this is a case where the simulation data need to be interpreted very carefully (and any assumptions stated very explicitly) because the physical system could be behaving very differently from the simulated system. Overall, I’m unconvinced of the conclusion above except for the shortest PFs.
4. Concern #3 then raises concerns about the conclusion on page 9 that “MT growth is limited by the rate of new tubulin attachments to the ends of protofilaments, not by the rate of lateral bond closure.” This is an interesting idea, but unless #3 is addressed in a robust way, I think that it needs to be stated much more tentatively.

To address concern 3 and 4 of the Reviewer, we have carried out a dedicated computational analysis of the dependence of a single protofilament fluctuation frequency on its length in the range of protofilament curl lengths spanning from 8 nm to 96 nm (new Fig. 1d). As suggested by the Reviewer, the frequency of protofilament fluctuations does depend on the protofilament curl length. But the number of passages from curved to straight shapes per second for *the lowest tubulin dimer* in the curved part of the protofilaments is still predicted to be very high, above 10^5^ Hz, even for protofilaments as long as 96 nm and having a relatively high bending stiffness (174 kcal/mol/rad^2^). This frequency is significantly higher than the rate of arrival and loss of tubulin subunits, confirming our previous conclusion that microtubule growth is not limited by the rate of the lateral bond closure.

5. A related issue is that I think that the authors need to incorporate into their discussions the rate of tubulin detachment in their simulations. More specifically, do the authors agree with previous papers that conclude that most incoming tubulin subunits leave before being incorporated into the MT? I assume that they do not. Some explicit discussion of (and perhaps data on) detachment rates would be useful.

The Reviewer is correct. We estimate tubulin on-rate to be about 0.6 s^-1^µM^-1^ per protofilament, relatively close to a recent direct measurement for yeast tubulin by Mickolajczyk et al., 2019, but about an order of magnitude lower than that proposed by Gardner et al., 2011. As the net MT growth is determined by the difference of the on- and off-rates, this means that our off-rates should also be significantly lower than proposed by Gardner and colleagues. We have added a comment about this in the last paragraph of page 10. However, we cannot exclude

that tubulins can form frequent, transient, unproductive encounters with MT tips. Our simulations did not take such events into account.

6. I’m unconvinced by the argument on Page 7 that when lateral activation energies are low, the model is “overly sensitive” to changes in lateral bond strength. Yes, the authors do use their simulations to show that when the activation energy is low, the system is very sensitive to changes in lateral bond strength. But who is to say that such sensitivity is incorrect? Biology is sometimes poised on a knife edge. Given the large number of regulatory proteins that affect tubulin lateral bond strength, one might even predict a sharp dependence on lateral bond strength.

It is our considered opinion that a high sensitivity of MT growth or depolymerization rate on the changes in any energetic parameter is unlikely, because in that case even small thermal fluctuations would lead to very significant variations in MT assembly and disassembly rates. However, our main argument for dismissing the low activation energy barrier is not based on the sensitivity of MT growth or shortening rate on the lateral bond strength, but rather on the inability of MT parameterized with a low activation barrier, to develop high forces during depolymerization (Fig 2f).

7. It would be helpful if the authors could compare their “raggedness” measurements to the “taper” measured by other authors.

We have added a clarification about the difference in the raggedness and taper in the last paragraph on page 12.

Other writing issues (mostly minor):

1. The legends are quite spare. It would be helpful if a bit more information could be provided. In particular, it would be helpful if each figure or figure panel could say the data come from.

We recognize the validity of the reviewer’s concern, but the space limitations defined by journal policy demand that we be as succinct as possible. We have looked at each legend and tried to revise it into the clearest form possible, given the need for verbal parsimony. Additionally, we have provided detailed simulation parameters in the Source Data file.

More details about tubulin concentration and other parameters would also be useful.

2. The methods and supplementary data have quite a few typos and/or problems with commas.

These sections need some editing to get them up to publication standards.

We have edited the supplementary data.

3. On page 25, please check the value for temperature in following sentence: “Simulations of the tubulin and Dam1 motions were carried out, using equation (9) at T = 0 K.”

Indeed, we used eq.9 with T set to zero in order to numerically solve the force balance and identify the maximal force, which a single curved PF could withstand against a given external load. Although a bit confusing, using eq. 9 with T = 0 is mathematically similar to application of

the gradient descent iterative minimization algorithm. We have added an explanatory note about this in Methods, to make this more clear.

Figures:

Figure 1: Nature Communications is a general science journal. Figure 1 should include a cartoon

showing how the protofilament visualized fits into the larger structure of a microtubule.

We have modified Figure 1a to show a PF in the context of the larger structure of MT.

Figure 3: panel D appears to be missing the Y axis label.
Fixed. Thank you.

**Reviewer comments, second version:**

Reviewer #1 (Remarks to the Author):

The authors have satisfactorily addressed my concerns.


Reviewer #2 (Remarks to the Author):

I thank the authors for addressing all my previous points. The article is now suitable for publication.


Reviewer #3 (Remarks to the Author):

Review of revised manuscript “Mechanisms of microtubule dynamics and force generation examined with computational modeling and electron cryotomography” by Gudimchuk et al.

Overall, I think that the authors have significantly improved the manuscript. I have a number of relatively minor additional concerns, all of which can be addressed at the level of writing:

My only significant concern is this: The new GTP hydrolysis work is a significant addition, but the authors need to clarify throughout the manuscript how the presence/absence of explicit GTP hydrolysis is being handled. More specifically, they should provide more detail about the following, even if it seems somewhat redundant to them: (a) whether explicit GTP hydrolysis was present for the simulations shown; (b) when explicit GTP hydrolysis was not present, which aspects of the simulation were altered to allow simulations without GTP hydrolysis to mimic the behavior of physical MTs with hydrolysis; (c) when it was present, which aspects of the simulation were altered and by how much for GTP vs. GDP tubulin.

Lack of clarity with regard to issue (b) leads to particular confusion in Figures 2F and 3B, where rapid shortening is presented for what appear to be GTP tubulin polymers. I assume that the answer lies in the fact that two different values are presented for the depth of the potential well for lateral bonds, but I did not find this explicitly stated anywhere. If a discussion of this point is present and I just missed it, I think that it needs to be stated more clearly.

Similarly, it is confusing that table S1 has a GTP hydrolysis rate constant, but no differentiation of GTP vs. GDP versions of any of the rate constants. As a result, when reading the main text, it is not clear how GTP hydrolysis is taken into consideration in the simulations. Page 21 and the legend to Figure 3 provide some explanation, but this point should be clarified both in the text and in the table of parameters, and the explanation should include how depolymerization in the GTP hydrolysis simulations relates to that that in simulations without GTP hydrolysis.

Minor:
1. On page 5: the text reads: “Deviations from the PF’s naturally curved shape incur bending deformations whose potential energies obey a simple Hookean law…”
Shouldn’t this be “are assumed to obey”?

2. On page 6, they state that they modified their older model, but the authors should make sure that they do explicitly state somewhere in what ways this simulation differs from the older model (perhaps I missed this).

3. Page 13: The authors state that “The model predicts that the extent of raggedness grows over time…” Could the authors please clarify over what time scale?

4. Page 17: The text states “Our model opens an additional possibility for accelerating MT
growth through favoring lateral bond formation between GTP-tubulin PFs where they meet in the MT wall. “ This is an important point, but it is not the first time such a suggestion has been made, and additional references might be helpful. For example, the cited Gardner et al (2011) made a very similar argument (though without the idea of non-laterally bound PFs), as did the cited Margolin et al (2012) paper (this time in the context of laterally unbound tip protofilaments).

**Author rebuttal, second version:**

Reviewer #3 (Remarks to the Author):

Review of revised manuscript “Mechanisms of microtubule dynamics and force generation examined with computational modeling and electron cryotomography” by Gudimchuk et al.

Overall, I think that the authors have significantly improved the manuscript. I have a number of relatively minor additional concerns, all of which can be addressed at the level of writing:

My only significant concern is this: The new GTP hydrolysis work is a significant addition, but the authors need to clarify throughout the manuscript how the presence/absence of explicit GTP hydrolysis is being handled.

More specifically, they should provide more detail about the following, even if it seems somewhat redundant to them: (a) whether explicit GTP hydrolysis was present for the simulations shown; (b) when explicit GTP hydrolysis was not present, which aspects of the simulation were altered to allow simulations without GTP hydrolysis to mimic the behavior of physical MTs with hydrolysis; (c) when it was present, which aspects of the simulation were altered and by how much for GTP vs. GDP tubulin.

We have added clarifications in the text (lines 153, 157-160, 222-223, 513) and in the legends to Figs 2, 4, 7, 8.

Lack of clarity with regard to issue (b) leads to particular confusion in Figures 2F and 3B, where rapid shortening is presented for what appear to be GTP tubulin polymers. I assume that the answer lies in the fact that two different values are presented for the depth of the potential well for lateral bonds, but I did not find this explicitly stated anywhere. If a discussion of this point is present and I just missed it, I think that it needs to be stated more clearly.

We extended figure legends to better explain model parameters. Specifically, we modified legends to Figs. 2f,g; 4a,b,c; 7b,c; 8a,b,c. The legend to Fig. 3b already had that information explicitly stated.

Similarly, it is confusing that table S1 has a GTP hydrolysis rate constant, but no differentiation of GTP vs. GDP versions of any of the rate constants. As a result, when reading the main text, it is not clear how GTP hydrolysis is taken into consideration in the simulations. Page 21 and the legend to Figure 3 provide some explanation, but this point should be clarified both in the text and in the table of parameters, and the explanation should include how depolymerization in the GTP hydrolysis simulations relates to that that in simulations without GTP hydrolysis.

We modified Supplementary Table 1 to clarify the parameters, which were used to describe GDP and GTP tubulins in the majority of the simulations and added a note to emphasize an alternative parametrization, which was additionally considered in Fig 3d.e.

Minor:

1. On page 5: the text reads: “Deviations from the PF’s naturally curved shape incur bending

deformations whose potential energies obey a simple Hookean law...”

Shouldn’t this be “are assumed to obey”?

We re-phrased that sentence to make it more clear.

2. On page 6, they state that they modified their older model, but the authors should make sure that they do explicitly state somewhere in what ways this simulation differs from the older model (perhaps I missed this).

We have explicitly stated the differences between the new and the previous model (lines 136­138).

3. Page 13: The authors state that “The model predicts that the extent of raggedness grows over time...” Could the authors please clarify over what time scale?

The raggedness in our model grows on the time scale of our simulations (a few seconds). We have stated that in line 299.

4. Page 17: The text states “Our model opens an additional possibility for accelerating MT growth through favoring lateral bond formation between GTP-tubulin PFs where they meet in the MT wall. “ This is an important point, but it is not the first time such a suggestion has been made, and additional references might be helpful. For example, the cited Gardner et al (2011) made a very similar argument (though without the idea of non-laterally bound PFs), as did the cited Margolin et al (2012) paper (this time in the context of laterally unbound tip protofilaments).

The studies, which the reviewer mentions, suggested a different mechanism of MT growth acceleration by MAPs. Although the model of Margolin et al., 2012 is conceptually similar to ours, its parametrization is closer to that of Gardner et al., 2011. In contrast to our model, the authors of both studies proposed that tubulin on and off rates were high. Therefore within those models the slow net MT growth could be accelerated by MAPs through decreasing the tubulin off-rate. Margolin et al., 2012 further specifically suggested that MAPs could be lowering the off-rate by promotion of lateral bonds (or ‘healing’ of lateral cracks).

Instead, we conclude that tubulin off-rate is fairly low, so decreasing it further would not significantly contribute to magnifying MT growth rate. Rather, MT growth rate in our model may be accelerated either by increasing the association rate of tubulins from solution or by increasing the rate of lateral bonding between adjacent curved PFs. Which pathway for MT acceleration is more efficient is determined by the relative contributions of these pathways under current conditions. For example, formation of lateral bonds is predicted to be very fast compared to tubulin on-rate in the absence of MAPs. Therefore, the most efficient way to accelerate MT growth in vitro is through increasing the tubulin on-rate, which is presumably done by XMAP215. EB1 becomes efficient only after the on-rate has been increased to the point that it becomes comparable with the rate of lateral bond formation. Hence the synergy of these proteins, which cannot be explained by the mechanisms proposed in the previous studies.

**Reviewer comments, third version:**

**Author rebuttal, third version:**
